# Structural insights into ribonucleoprotein dissociation by nucleocapsid protein interacting with non-structural protein 3 in SARS-CoV-2

**DOI:** 10.1038/s42003-023-04570-2

**Published:** 2023-02-18

**Authors:** Xincheng Ni, Yinze Han, Renjie Zhou, Yanmei Zhou, Jian Lei

**Affiliations:** 1 National Clinical Research Center for Geriatrics, West China Hospital, Sichuan University, Chengdu, Sichuan China; 2 State Key Laboratory of Biotherapy and Cancer Center, West China Hospital, Sichuan University, Chengdu, Sichuan China

**Keywords:** X-ray crystallography, Virology

## Abstract

The coronavirus nucleocapsid (N) protein interacts with non-structural protein 3 (Nsp3) to facilitate viral RNA synthesis and stabilization. However, structural information on the N-Nsp3 complex is limited. Here, we report a 2.6 Å crystal structure of the N-terminal domain (NTD) of the N protein in complex with the ubiquitin-like domain 1 (Ubl1) of Nsp3 in severe acute respiratory syndrome coronavirus 2 (SARS-CoV-2). One NTD and two Ubl1s formed a stable heterotrimer. We performed mutational analysis to reveal the key residues for this interaction. We confirmed the colocalization of SARS-CoV-2 N and Nsp3 in Huh-7 cells. N-Ubl1 interaction also exists in SARS-CoV and Middle East respiratory syndrome coronavirus. We found that SARS-CoV-2 Ubl1 competes with RNA to bind N protein in a dose-dependent manner. Based on our results, we propose a model for viral ribonucleoprotein dissociation through N protein binding to Ubl1 of Nsp3.

## Abstract (web summary)

Reporting the crystal structure of the N-terminal domain of the viral nucleocapsid protein in complex with the ubiquitin-like domain 1 of Nsp3 in SARS-CoV-2

## Introduction

Severe acute respiratory syndrome coronavirus 2 (SARS-CoV-2) and its variants pose a threat to public health systems, continuously leading to the global coronavirus disease 2019 (COVID-19) pandemic. Although vaccines and several drugs have been utilized to fight against SARS-CoV-2^1–5^, the incidence of new infection cases is increasing rapidly. Therefore, it is necessary to investigate the basic principles of coronavirus replication to identify new possible targets for antiviral therapies.

SARS-CoV-2 is the third highly-pathogenic human coronavirus in the past two decades, the first two being SARS-CoV and Middle East respiratory syndrome coronavirus (MERS-CoV)^6,7^. SARS-CoV-2 is an enveloped positive-sense single-stranded RNA (+ssRNA) virus belonging to the genus *β*-coronavirus in the family *Coronaviridae*. SARS-CoV-2 possesses a genome of approximately 30 kb in size. The 5′-terminal two-thirds of its genome encodes two overlapping polyproteins, pp1a and pp1ab. These polyproteins are further digested by two viral proteases to generate 16 individual non-structural proteins (Nsps) in total, which are essential for the viral replication/transcription complex (RTC) formation^8^. While the 3′-proximal one-third of the SARS-CoV-2 genome encodes a set of accessory proteins and four structural proteins (spike (S), envelope (E), membrane (M), and nucleocapsid (N)).

CoV-N protein is a multifunctional protein that plays a crucial role in the viral life cycle^9,10^. It exhibits a highly conserved architecture in all four genera of coronaviruses. The N protein comprises the N-terminal domain (N-NTD) and the C-terminal domain (N-CTD), sandwiched by three intrinsically disordered regions (IDRs), namely the N-terminal arm (N-arm), the central linker region (LKR) containing the Ser/Arg (SR)-rich motif, and the C-terminal tail (C-tail)^11^. The fundamental function of the N protein is to interact with viral genomic RNA to form a helical ribonucleoprotein (RNP) complex^10^, which facilitates viral transcription and assembly. Also, the N protein was identified to assist viral replication and infectivity through liquid-liquid phase separation (LLPS) in SARS-CoV-2^12–15^. LLPS is mediated by its IDRs and RNA binding^12,13^. Furthermore, the N protein displays extensive protein-protein interactions (PPIs) with host and viral proteins. For example, the N protein can bind several components (such as G3BP-1/2 (GTPase-activating protein SH3 domain-binding protein 1/2) and hnRNPs (heterogeneous nuclear ribonucleoproteins)) of host stress granules to induce LLPS^14,15^. It can also interact with host NLRP3 (NLR family PYRIN domain containing-3) to prompt hyperinflammation^16^. Meanwhile, N protein shows the ability to interact with viral M protein for viral assembly^17^. In particular, the N protein interacts with Nsp3 to facilitate viral replication and infectivity^18–25^.

In coronaviruses, Nsp3 is a trans-endoplasmic reticulum (ER) membrane protein with multiple domains^26^. It is essential for viral RTC^27^ and double-membrane vesicles (DMVs) formations^28^. The mouse hepatitis virus (MHV) N protein is recruited to RTC via direct interaction with Nsp3, but not other Nsps, which is critical for viral RNA synthesis^24^. The impaired N-Nsp3 interaction inhibits viral RNA transcription and replication in different CoVs^22–24,29^. Using cryo-electron microscopy, Wolff et al. observed that hexameric Nsp3 of MHV forms the core of a molecular pore on the DMV membrane, leading to the export of newly produced viral genomic RNA to the cytosol by interacting with N protein^30^. However, the exact operating mode of this molecular pore is unknown, and detailed information on the relevance of Nsp3, N, and RNA around DMVs is lacking. High-resolution structural information on either N-RNA or N-Nsp3 (including parts of DMV) is helpful to address these questions; however, such structures are scarce. To the best of our knowledge, only SARS-CoV-2 NTD with ssRNA/double-stranded RNA (dsRNA)^31,32^ and SARS-CoV-2 N-LKR region in complex with parts of ubiquitin-like domain 1 (Ubl1) (residues 16–111, N-terminal domain of Nsp3)^33^, have been recently reported. In the latter structure, Bessa et al. found that two liner motifs of N-LKR bind to Ubl1 with tens of nanomolar range affinity^33^. Additionally, they mentioned that probable domain contacts exist between N-NTD and Ubl1 according to the small-angle X-ray scattering (SAXS) assay. Keane & Giedroc also reported NTD could interact with Ubl1 of Nsp3 in MHV^34^. Thus, NTD is likely to be the other binding site for Ubl1.

In order to expand the structural information on N-Nsp3 interaction and further understand how this interaction contributes to viral RNA replication, we determined the crystal structure of N-NTD in complex with two copies of Ubl1 (residues 1–111) of Nsp3 in SARS-CoV-2. The NTD-Ubl1 heterotrimer was confirmed by cross-linking and dynamic light scattering (DLS) assays. The key residues for NTD-Ubl1 interactions were unveiled. Notably, SARS-CoV-2 Ubl1 and a transcriptional regulatory sequence (TRS) RNA compete with each other for interacting with N protein. Based on our results, we generated a possible model for viral RNP dissociation that is regulated by the interactions between the N protein and Ubl1 of Nsp3. Structural information on such interactions could provide new strategies for antiviral therapies.

## Results

### SARS-CoV-2 N protein colocalizes with Nsp3 in Huh-7 cells

**Fig. 1:**
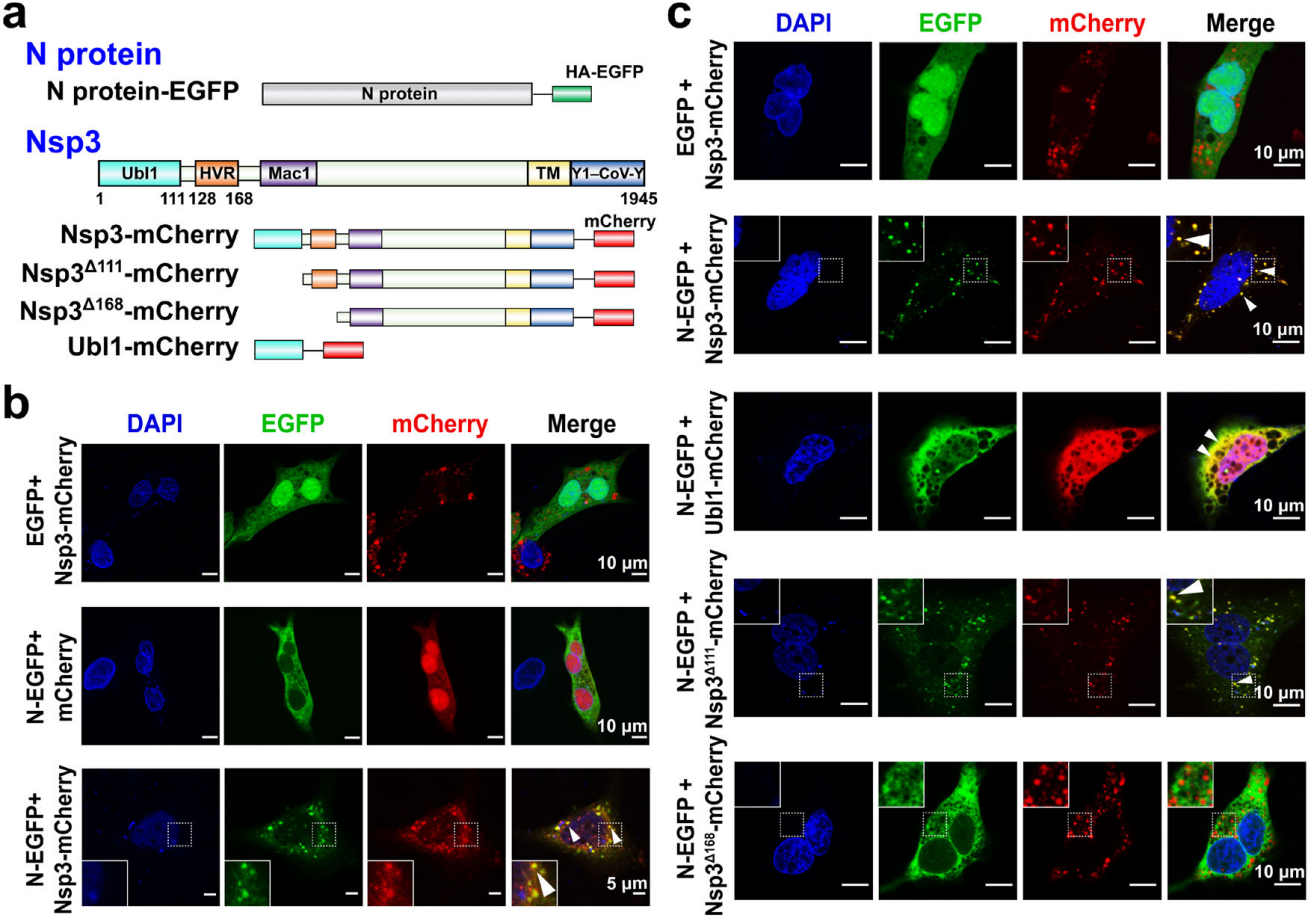
SARS-CoV-2 N protein and Nsp3 colocalize in Huh-7 cells. **a** Schematic representation of the N protein as well as Nsp3, Nsp3^Δ111^, Nsp3^Δ168^, and Ubl1 expression vectors. Nsp3 non-structural protein 3, Ubl1 ubiquitin‐like domain 1, HVR hypervariable region, Mac1 macrodomain 1, TM transmembrane domain, Y1–CoV-Y domains of Y1 and CoV-Y. **b** Colocalization (see white-triangle arrows) between N protein and Nsp3 in Huh-7 cells. Localization of N protein and Nsp3 were analyzed with fluorescence labeling and viewed at 48 h post-transfection using an Olympus Spin co-focus fluorescence microscope. **c** Nsp3 colocalizes with N protein through its Ubl1 and HVR regions in Huh-7 cells. Nsp3^Δ111^: the deletion of Ubl1 (residues 1–111) in Nsp3; Nsp3^Δ168^: the deletion of Ubl1–HVR (residues 1–168) in Nsp3.

Previous studies have proved that N-Nsp3 interaction exists in various CoVs (such as MHV, bovine coronavirus (BCoV), SARS-CoV, and SARS-CoV-2^20,23,29^). We initially investigated the interaction between the SARS-CoV-2 N protein and Nsp3 in cells. N protein vectors with an enhanced green fluorescent protein (EGFP) tag and Nsp3 with mCherry (a red fluorescent protein tag, Fig. 1a) were expressed in Huh-7 cells. The colocalization of N and Nsp3 was identified in the cytosol of Huh-7 cells (Fig. 1b), indicating an interaction between the N protein and Nsp3.

### The Ubl1 and HVR regions of Nsp3 interact with the N protein

Ubl1 (ubiquitin-like domain 1) and the HVR (hypervariable region) are located at the N-terminus of Nsp3 (Fig. 1a). The former domain is essential for Nsp3 binding to the N protein in MHV and BCoV^22,23^. Therefore, we first examined the interaction between the Ubl1 domain of Nsp3 and N protein in SARS-CoV-2. As shown in Fig. 1c, the colocalization of Ubl1 and the N protein was confirmed in Huh-7 cells. However, unlike full-length Nsp3, Ubl1 was diffusely distributed in the cytosol of Huh-7 cells. This could be due to the loss of the C-terminal transmembrane domain (TM) of Nsp3 (Fig. 1a), leading to impaired localization of Nsp3 in the ER.

To determine if Ubl1 is the only region of Nsp3 that interacts with the N protein, we deleted the Ubl1 domain (N-terminal 111 residues) of Nsp3 (designated Nsp3^Δ111^). However, truncated Nsp3^Δ111^ could still interact with the N protein, although the N-Nsp3^Δ111^ interaction was attenuated compared to that of N and intact Nsp3 (Fig. 1c). These results indicate that other region(s) of Nsp3 is (are) involved in binding to the N protein. We next removed the adjacent HVR region, in addition to Ubl1 (designated Nsp3^Δ168^). The colocalization of Nsp3^Δ168^ and N protein was completely abolished (Fig. 1c). Taken together, these results suggest that the two N-terminal regions of Nsp3 (Ubl1 and HVR) mediate its binding to N protein.

### The NTD and LKR regions of N protein bind to Ubl1 of Nsp3

**Fig. 2:**
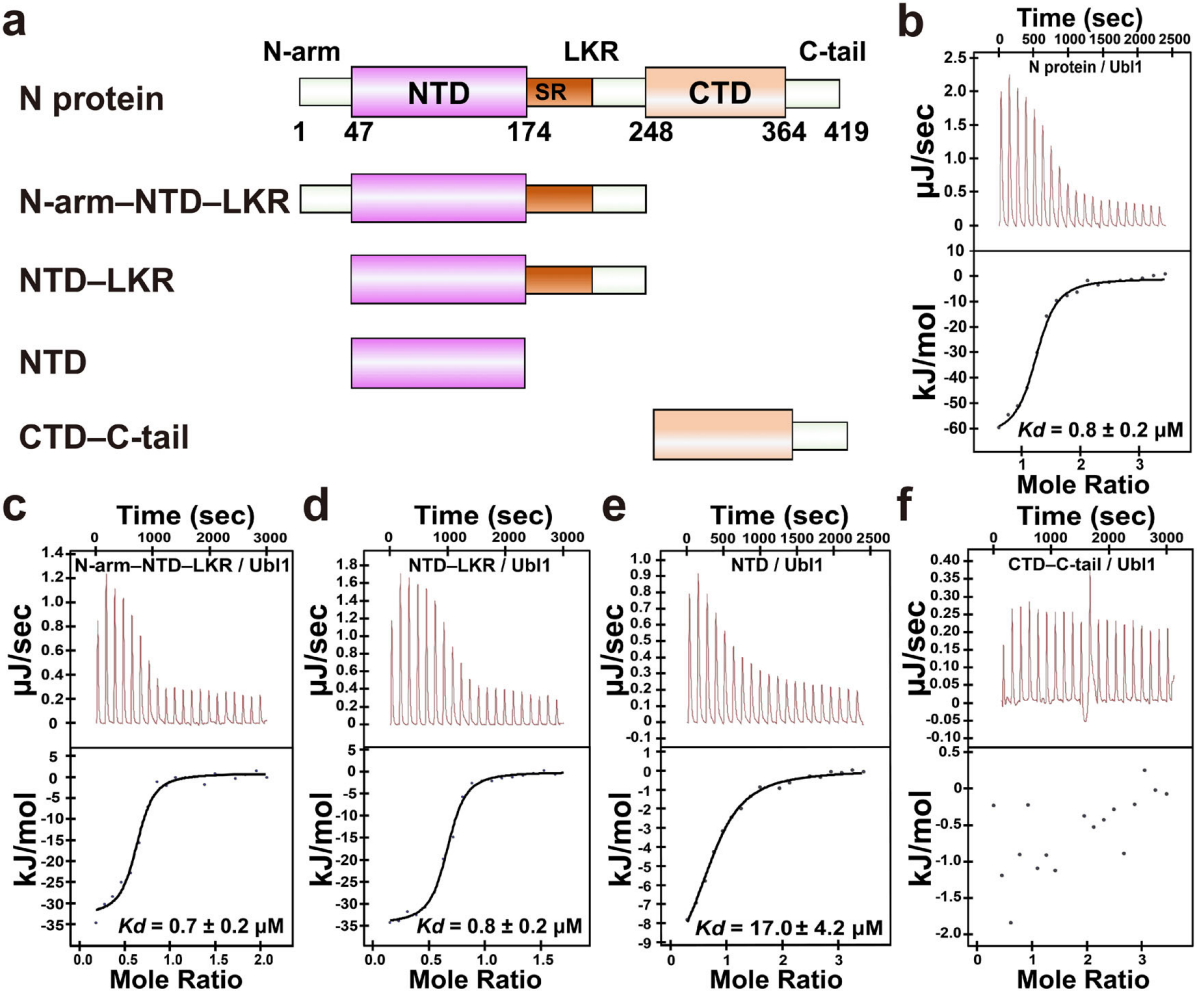
ITC measurement of the binding affinity between SARS-CoV-2 N and Ubl1. **a** Schematic diagram of SARS-CoV-2 N and its truncations. N-arm N-terminal arm, NTD N-terminal domain, LKR the central linker region, SR Ser/Arg-rich motif, CTD C-terminal domain, C-tail C-terminal tail. **b**–**f** Affinity between N protein (**b**) N-arm–NTD–LKR (**c**) NTD–LKR (**d**) NTD (**e**) CTD–C-tail (**f**) and Ubl1, respectively. The raw calorimetric curve is shown in the top panel, while the fitted binding isotherm curve is displayed in the bottom panel. *Kd*: dissociation constant.

The SARS-CoV-2 N protein comprises five distinct regions: two folded NTD and CTD domains separated by three IDRs, namely the N-arm, the LKR (including the SR-rich motif), and the C-tail (Fig. 2a). To verify the Nsp3-binding regions of the N protein, we prepared a series of truncated N proteins (Fig. 2a) and assessed their binding affinity with Nsp3 by isothermal titration calorimetry (ITC) assay. We used Ubl1 instead of Ubl1–HVR or full-length Nsp3 to perform this assay, because the latter two proteins were either easily degraded or difficult to obtain in sufficient quantity. The direct interaction between the N protein and Ubl1 had a dissociation constant (*Kd*) of 0.8 ± 0.2 μM (Fig. 2b and Table 1). The binding affinities between Ubl1 and N-arm–NTD–LKR, NTD–LKR, and the NTD alone had *Kd*s of 0.7 ± 0.2 μM, 0.8 ± 0.2 μM, and 17.0 ± 4.2 μM, respectively (Fig. 2c–e and Table 1). NTD–LKR exhibited nearly the same binding ability to Ubl1 as the intact N protein (Fig. 2b, d). Compared to NTD–LKR, NTD alone showed an approximately 20-fold reduction in binding affinity to Ubl1 (Fig. 2d, e). In addition, no appreciable interaction was observed between Ubl1 and the CTD–C-tail (Fig. 2f). Collectively, these results indicate that the NTD and LKR regions of the N protein are essential for interacting with Ubl1, while the CTD and C-tail are not. The interaction pattern between LKR and Ubl1 has recently been reported^33^. Thus, it is interesting to investigate the binding fashion between NTD and Ubl1.

**Table 1:** Thermodynamic parameters of the binding between N and their cognate Ubl1 in three CoVs.

Substrate	Ligand	*Kd* (μM)	*n*	ΔH (kJ/mol)	ΔS (J/mol·K)
**SARS-CoV-2**					
N	Ubl1	0.8 ± 0.2	1.19 ± 0.02	−62.5 ± 2.6	−99.0
Ubl1	N-arm–NTD–LKR	0.7 ± 0.2	0.60 ± 0.02	−33.7 ± 1.9	0.9
Ubl1	NTD–LKR	0.8 ± 0.2	0.64 ± 0.01	−34.5 ± 1.2	−2.5
Ubl1	NTD	17.0 ± 4.2	0.73 ± 0.03	−11.3 ± 1.2	52.3
Ubl1	CTD–C-tail	N.D.^a^	-	-	-
Ubl1	NTD R92A	55.7 ± 16.3	0.58 ± 0.11	−13.3 ± 3.2	35.5
Ubl1 *E26A*	NTD	78.6 ± 25.3	0.74 ± 0.04	−14.1 ± 1.7	29.7
Ubl1 *E95A*	NTD	53.1 ± 18.2	0.75 ± 0.08	−5.2 ± 1.2	63.8
Ubl1 *Y103A*	NTD	57.7 ± 22.4	0.57 ± 0.05	−3.1 ± 0.6	70.5
Ubl1 *D110A*	NTD	50.8 ± 14.3	0.64 ± 0.06	−11.1 ± 0.8	44.0
**SARS-CoV**					
N	Ubl1	0.8 ± 0.2	1.00 ± 0.02	−55.9 ± 1.6	−76.2
**MERS-CoV**					
N	Ubl1	2.3 ± 0.6	0.82 ± 0.02	25.5 ± 1.3	196.0

^a^N.D.: not determined.

### Preparation of the NTD-Ubl1 complex protein in vitro

**Fig. 3:**
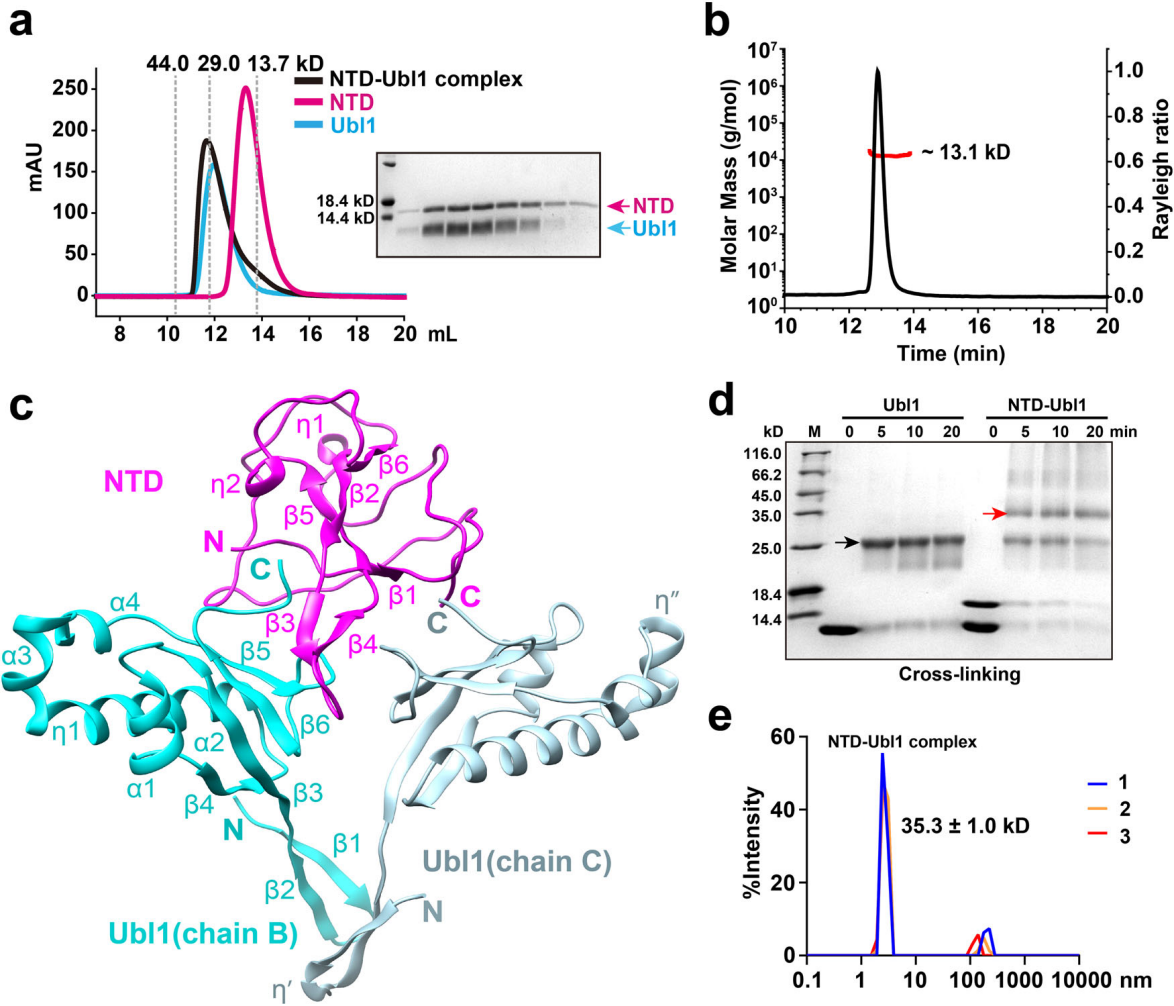
Crystal structure of SARS-CoV-2 NTD in complex with Ubl1. **a** The NTD-Ubl1 complex protein was confirmed by SEC assay. NTD: ~14.3 kD. Ubl1: ~12.9 kD. NTD-Ubl1 complex: ~40.1 kD. The peak positions of three standard proteins are shown as gray dashed lines. **b** SEC-MALS analysis of Ubl1 in solution. The light scattering data is displayed in a black curve and the molar mass data is shown in a red curve. The calculated MW of Ubl1 is ~13.1 kD. **c** The crystal structure of the NTD in complex with Ubl1. NTD is shown in magenta, and two Ubl1s are displayed in cyan (chain B) and light blue (chain C). The N- and C- termini of NTD and Ubl1s are marked in the corresponding colors. Compared to chain-B Ubl1, the two extra 3_10_ helices in chain-C Ubl1 are labeled as η′ and η″. **d** Cross-linking experiments of Ubl1 as well as NTD-Ubl1 complex. The compositions of the samples after cross-linking were identified by SDS-PAGE. Bands corresponding to the dimeric Ubl1 (theoretical MW: ~25.8 kD) and the heterotrimeric NTD-Ubl1 complex (~40.1 kD) are indicated by black and red arrows, respectively. **e** Dynamic light scattering analysis of the NTD-Ubl1 complex in three independent experiments (*n* = 3). Radius: ~2.7 nm; Calculated MW: 35.3 ± 1.0 kD. Figure **c** was prepared using Chimera (http://www.cgl.ucsf.edu/chimera/).

SARS-CoV-2 NTD and Ubl1 were separately purified by size-exclusion chromatography (SEC). The NTD and Ubl1 were subsequently mixed at a molar ratio of ~1:2 overnight (considering that the binding stoichiometry (n) between these two proteins was about 0.7 in the ITC assay, Table 1). Then the mixture was subjected to an SEC experiment the next day. The peak position of this mixture was shifted left compared to that of the NTD and Ubl1 alone, in addition, the presence of NTD and Ubl1 in the elution fraction derived from this mixture peak was confirmed by SDS-PAGE (Fig. 3a). Taken together, these results imply the formation of the NTD-Ubl1 complex protein in vitro.

During the purification of Ubl1 (theoretical molecular mass (MW) 12.9 kD), a significantly lower elution volume was observed in our SEC (Fig. 3a). Serrano et al. previously reported a similar observation on the purification of SARS-CoV Ubl1^35^. They confirmed that SARS-CoV Ubl1 is indeed a monomer and explained that the elongated (not spherical) shape of Ubl1 likely leads to a lower retention volume in the SEC^35^. To investigate whether SARS-CoV-2 Ubl1 possesses the same oligomerization status, we performed an SEC-coupled to multi-angle light scattering (SEC-MALS) assay. The calculated MW of Ubl1 is about 13.1 kD (Fig. 3b), indicating that SARS-CoV-2 Ubl1 also exists as a monomer.

### Crystal structure of the N-NTD in complex with Ubl1

To elucidate the structural basis of the interactions between the NTD and Ubl1, we determined the crystal structure of SARS-CoV-2 NTD in complex with Ubl1 at a resolution of 2.6 Å (PDB code: 7WZO). The crystallographic statistics are presented in Table 2. The overall structure of the NTD-Ubl1 complex comprises an intermediate NTD monomer wrapped by two Ubl1s forming a “V-like” shape (Fig. 3c; two Ubl1s are designated as chain-B and chain-C Ubl1, respectively). According to the DSSP server^36^, the NTD molecule displays secondary structural elements in the order β1–η1–β2–β3–β4–β5–η2–β6 (η: 3_10_ helix). The core antiparallel β‐sheet (β6–β2–β5–β1) is flanked by two short 3_10_ helices and loops. A protruding β-hairpin (β3–β4) of the NTD inserts into the concave area of the “V-like” shape formed by two Ubl1s (Fig. 3c). The interface areas of the NTD with chain-B and chain-C Ubl1 are approximately 880 and 850 Å^2^, respectively, calculated through the PDBePISA server^37^. The conformation of the NTD in the complex is very close to that of the NTD alone (PDB code: 7VNU). The root-mean-square difference (RMSD) between these two conformations is about 0.5 Å for all the Cα atoms.

**Table 2:** Data collection and refinement statistics.

	NTD-Ubl1 complex	NTD
**Data collection**		
Space group	*P3*_*1*_*21*	*P2*_*1*_*2*_*1*_*2*_*1*_
Cell dimensions		
*a*, *b*, *c* (Å)	69.54, 69.54, 269.25	58.71, 91.41, 96.12
*α*, *β*, *γ* (°)	90.00, 90.00, 120.00	90.00, 90.00, 90.00
Resolution (Å)	20.02-2.64 (2.77-2.64)	19.57-1.95 (2.00-1.95)
R_merge_	0.150 (1.187)	0.050 (0.698)
Mean *I*/σ(*I*)	13.2 (2.4)	19.8 (2.6)
Unique reflections	23,164 (3,003)	38,346 (2,633)
Completeness (%)	99.7 (99.3)	99.8 (100.0)
Redundancy	18.7 (15.3)	6.5 (6.7)
**Refinement**		
Resolution (Å)	20.02-2.64	19.57-1.95
*R*_factor_/*R*_free_	0.214/0.246	0.188/0.226
No. atoms		
Protein	2,728	3,992
Water	118	541
*B*-factors		
Protein	71.4	39.9
Water	74.9	44.4
R.m.s. deviations		
Bond lengths (Å)	0.009	0.010
Bond angles (°)	1.084	1.597
**PDB code**	7WZO	7VNU

Values in parentheses are for the highest resolution shell.

$${R}_{{{{{{\rm{factor}}}}}}}=\,{\sum }_{{hkl}}\left|{F}_{o}\left({hkl}\right)-{F}_{c}\left({hkl}\right)\right|/{\sum }_{{hkl}}{{|F}}_{o}\left({hkl}\right)|$$.

*R*_free_ was calculated for a test set of reflections (~ 5.0%) omitted from the refinement.

In the NTD-Ubl1 ternary complex, each Ubl1 monomer consists of a long N-terminal β-hairpin region (β1–β2, Ala1–Val15), as well as a typical ubiquitin-like fold region, with secondary structural elements in the order β1–β2–β3–α1–β4–α2–η1–α3–α4–β5–β6 (Fig. 3c). In contrast, the N-terminal 15 residues of the SARS-CoV-2 Ubl1 present highly flexible conformations in recent NMR studies^33,38^. The overall conformation of Ubl1 chains B and C is almost identical. The RMSD between these two Ubl1s is about 0.3 Å for all the Cα atoms. The main differences are the two additional 3_10_ helices (η′ and η″) in chain C (Fig. 3c). We observed that two Ubl1s contact with each other. The interface area of these two Ubl1s is ~240 Å^2^. The main interaction regions are located at the N-terminal β-hairpin as well as the loop between β5 and β6 at the C terminus of two Ubl1s.

To verify whether the NTD binding to Ubl1 in a molar ratio 1:2 exists in solution, we performed cross-linking assays of free Ubl1 and the NTD-Ubl1 complex, respectively (Fig. 3d). The free Ubl1 forms a stable homodimer (theoretical MW is 25.8 kD) but no other multimers. This result could support close contacts exist between two Ubl1s in solution. Meanwhile, we observed a newly generated ~35 kD band in the cross-linking assay of the NTD-Ubl1 complex (Fig. 3d). The amount of this band was increased along with the decreased amounts of the NTD monomer and Ubl1 dimer (see the last right three lanes in Fig. 3d). We suspected that the ~35 kD bands were likely formed by one NTD interacting with the Ubl1 dimer (the theoretical MW of this ternary complex is about 40 kD). Moreover, we performed a dynamic light scattering (DLS) assay to evaluate the MW of the NTD-Ubl1 complex in solution. The calculated MW of this complex is 35.3 ± 1.0 kD (Fig. 3e), which also supports the 1:2 binding molar ratio between NTD and Ubl1 likely exits in solution.

### Ionic interaction is the dominant binding pattern for NTD interacting with Ubl1

**Fig. 4:**
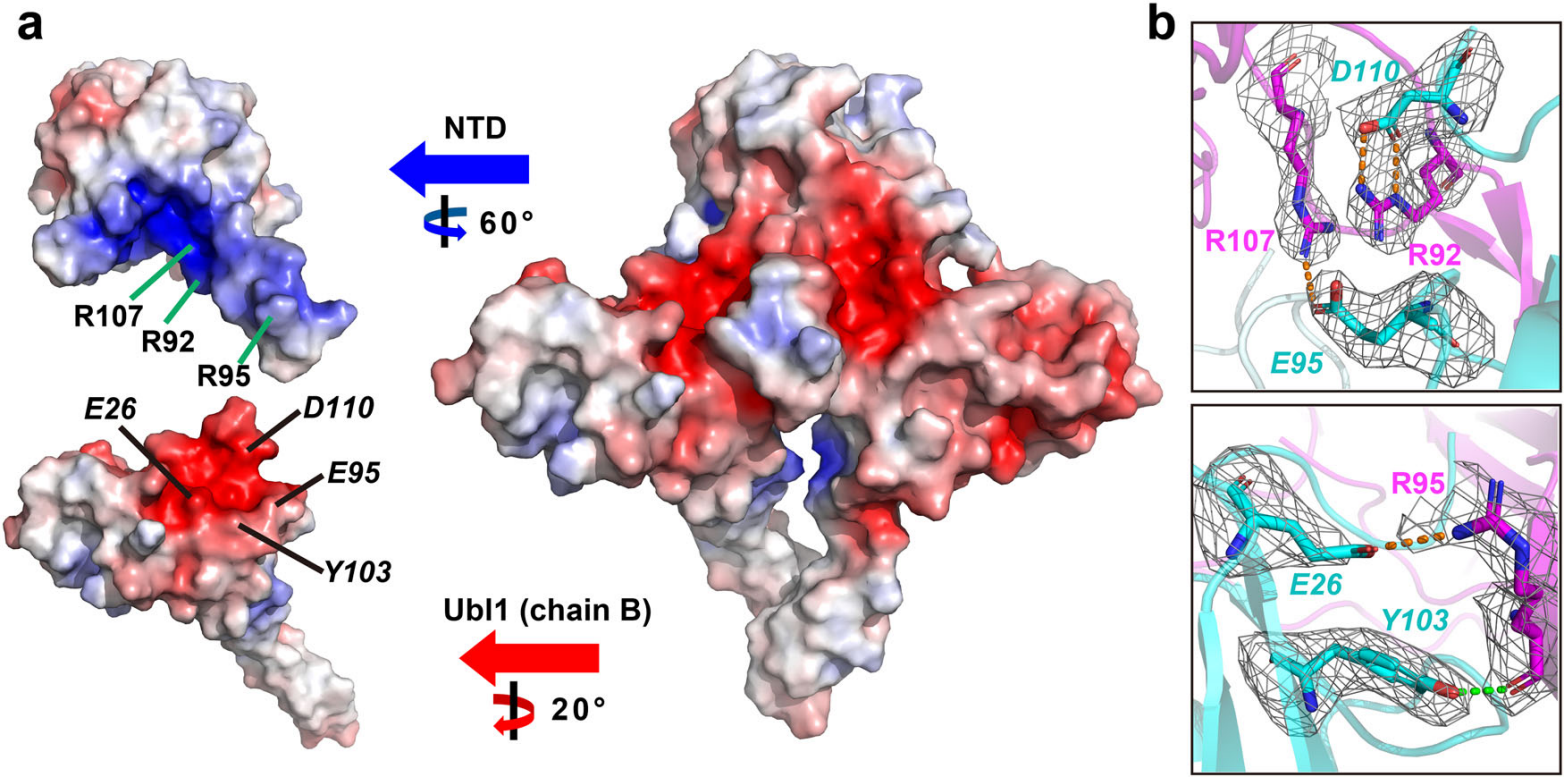
Interaction mode of SARS-CoV-2 NTD binding to Ubl1. **a** The electrostatic surface (−5 kBT/e (red) – +5 kBT/e (blue)) of the NTD-Ubl1 complex. Residues R92, R95, and R107 of NTD comprise the positively charged center. Residues *E26*, *E95*, and *D110* of Ubl1 form the negatively charged center. **b** Three salt bridges R92-*D110*, R95-*E26*, R107-*E95*, and one hydrogen bond (R95---*Y103*) are shown. *Fo* – *Fc* density maps (σ = 2.5; gray mesh) for these residues are displayed. Figures (**a**, **b**) were prepared using PyMOL (https://pymol.org).

The interaction surfaces between the NTD and two Ubl1s display excellent complementarity (Fig. 4a). The major interaction regions are located at the β-hairpin (β3–β4) of NTD as well as at the C-terminus (around β5–β6) of two Ubl1s (Fig. 3c). The predominant interaction mode is the ionic interaction between the two proteins (Fig. 4a). The positively charged β-hairpin of the NTD inserts into the negatively charged area formed by two Ubl1s. Arg92, Arg95, and Arg107 of the NTD β-hairpin comprise the positively charged center. While *Glu26*, *Glu95*, and *Asp110* (Ubl1 residues are in italics) of Ubl1 form the negatively charged center. Particularly, Arg92, Arg95, and Arg107 are also potential nucleic acid-binding sites of NTD in SARS-CoV-2^31^. Three salt bridges (Arg92-*Asp110*, Arg95-*Glu26*, and Arg107-*Glu95*, Fig. 4b) exist between the NTD and chain-B Ubl1. To investigate how these salt bridges affect the binding between the two proteins, we prepared a series of point mutations to disrupt these ionic interactions. Arg92Ala or *Asp110Ala* mutations reduced the binding affinity by about 3.3- or 3.0-fold between NTD and Ubl1, respectively (Table 1 and Supplementary Fig. 1). Furthermore, *Glu26Ala* or *Glu95Ala* mutations reduced the binding ability by ~4.6- or 3.1-fold (Table 1 and Supplementary Fig. 1). These results suggest that each of the three salt bridges is important for NTD binding to Ubl1.

In addition to the main ionic interactions, the main-chain oxygen atom of Arg95 in the NTD forms a hydrogen bond with the side chain of *Tyr103* in chain-B Ubl1 (Fig. 4b). The side chain of Arg107 in NTD and the side chain of *Ser93* in chain-C Ubl1, as well as the side chain of Ser105 in NTD and the backbone oxygen of *Asp110* in chain-C Ubl1, make up an additional two hydrogen bonds. Among the three mentioned hydrogen bonds, we found that *Tyr103Ala* mutation diminished the binding affinity by ~3.4-fold (Table 1 and Supplementary Fig. 1), indicating the importance of this bond. Moreover, we found that the key residues Arg92, Arg95, and Arg107 of the NTD and *Glu26*, *Glu95*, *Asp110*, and *Tyr103* of Ubl1 were absolutely conserved among SARS-CoV-2 and its variant strains (such as the delta and omicron strains) (Supplementary Fig. 2). Taken together, these results indicate that ionic interactions are the predominant binding mechanism between NTD and Ubl1. In addition, this conserved ionic interaction mode is present in all current SARS-CoV-2 variants.

### The N-Nsp3 interaction exists in other two human pathogenic coronaviruses in vitro

**Fig. 5:**
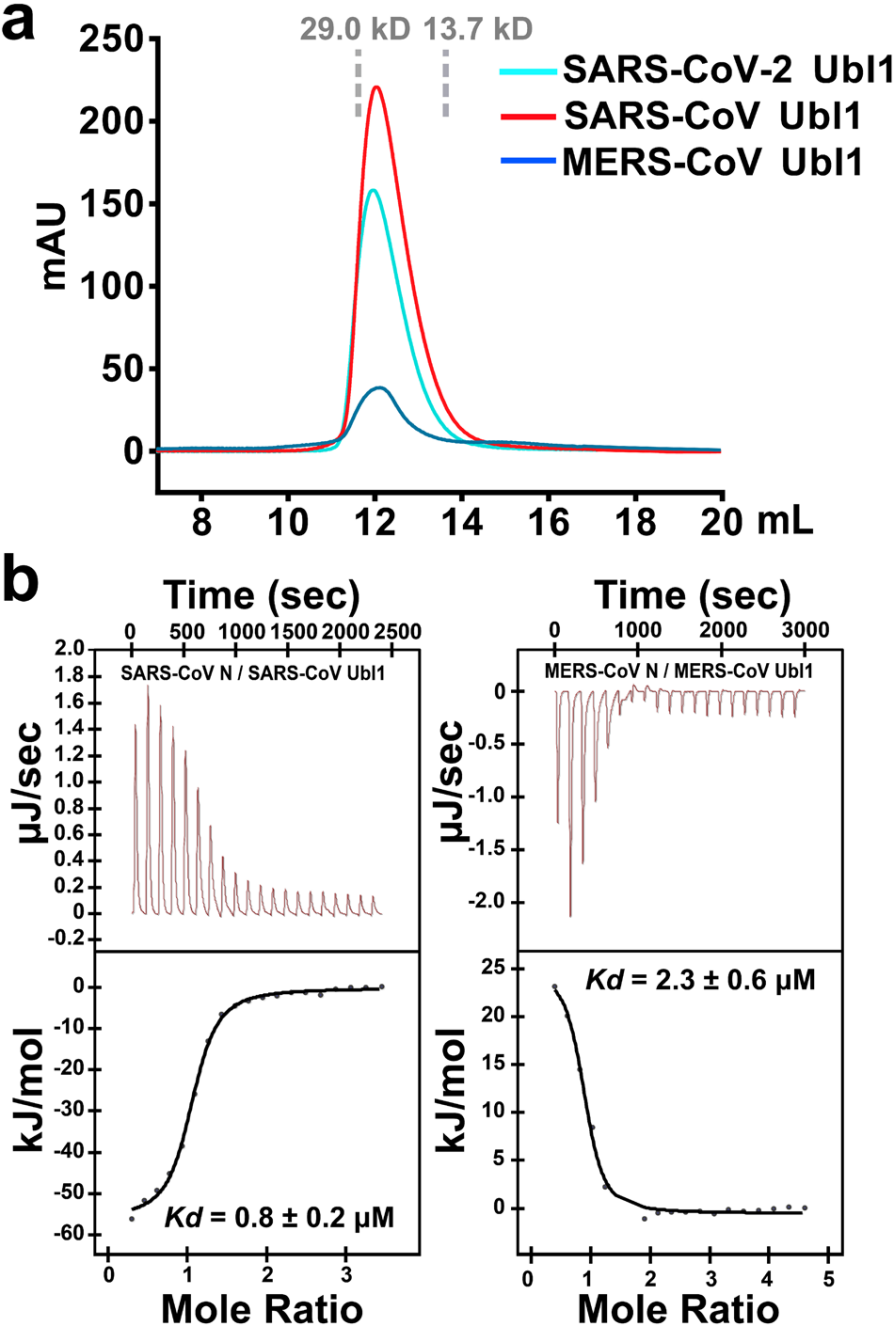
The N-Nsp3 interaction exists in SARS-CoV and MERS-CoV. **a** The Ubl1s of SARS-CoV, MERS-CoV, and SARS-CoV-2 present the lower elution volumes in SEC assays. The peak positions of two standard proteins (13.7 and 29.0 kD) are shown as gray dashed lines. **b** Affinity measurements of N and Ubl1 in SARS-CoV and MERS-CoV.

Before the appearance of SARS-CoV-2, the most recent highly-pathogenic human CoVs were SARS-CoV and MERS-CoV^6,7^. Previous studies have shown that the N-Nsp3 interaction exists in SARS-CoV^20^. However, to the best of our knowledge, no MERS-CoV N-Nsp3 interaction had previously been reported. In the present study, we purified the corresponding Ubl1 and N proteins from SARS-CoV and MERS-CoV. Consistent with SARS-CoV and SARS-CoV-2 Ubl1s, the Ubl1 of MERS-CoV presented a lower elution volume in the SEC assay (Fig. 5a). Next, we determined the dissociation constants between N and Ubl1 in SARS-CoV and MERS-CoV using ITC. The binding affinities of N-Ubl1 in SARS-CoV and MERS-CoV are about 0.8 and 2.3 μM, respectively (Fig. 5b and Table 1). Interestingly, the interaction between MERS-CoV N and Ubl1 was endothermic according to the ITC assay (Fig. 5b). The almost identical binding affinities of N and Ubl1 in SARS-CoV and SARS-CoV-2 are expected because of the high homology (~90% sequence identity) between these two viruses (Supplementary Fig. 3). In addition, key residues Arg92, Arg95, and Arg107 in the NTD and *Glu26*, *Glu95*, *Tyr103*, and *Asp110* of Ubl1, which are involved in SARS-CoV-2 NTD-Ubl1 binding, are absolutely conserved between SARS-CoV-2 and SARS-CoV (Supplementary Fig. 3). On the other hand, these key residues in SARS-CoV/SARS-CoV-2, correspond to residues Arg83, Asn86, and Arg97 in NTD and *Asn27*, *Asp97*, *Ile105*, and *Glu112* in Ubl1 of MERS-CoV (Supplementary Fig. 3). Thus, the presence of Asn86 and *Asn27* in MERS-CoV lead to the loss of the corresponding Arg95-*Glu26* salt bridge in SARS-CoV/SARS-CoV-2. The presence of *Ile105* (*Tyr103* in SARS-CoV/SARS-CoV-2) could damage the important hydrogen bond *Tyr103---*Arg95 in the MERS-CoV NTD-Ubl1 interaction. These mutations could partially explain the lower binding affinity of MERS-CoV N interacting with Ubl1. Collectively, the N-Nsp3 interaction exists in SARS-CoV, MERS-CoV, and SARS-CoV-2 *in* vitro. Whether the different binding affinities of N-Nsp3 in these CoVs are related to any biological function merits further investigation.

### Ubl1 and RNA compete with each other for N protein binding

**Fig. 6:**
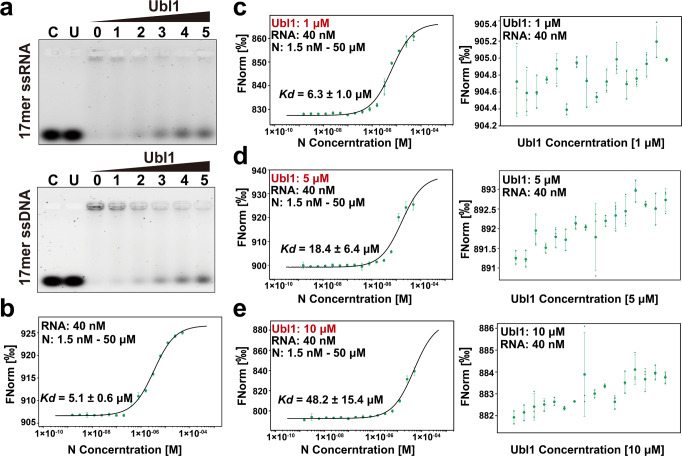
SARS-CoV-2 Ubl1 inhibits N protein binding to RNA. **a** Ubl1 disrupts N-RNA binding in a dose-dependent manner according to EMSA assay. Lane C: ssRNA/ssDNA; Lane U: Ubl1 + ssRNA/ssDNA, Lane 0: N + ssRNA/ssDNA. Lanes 1–5, ssRNA/ssDNA plus different molar ratios of Ubl1 to N protein (from 1:5 to 5:5). The amount of free ssRNA/ssDNA from the N-RNA complex increases with increasing concentrations of Ubl1 in Lanes 1–5. **b** Curve fit of MST traces for determination of the N-ssRNA binding affinity. **c**–**e** The binding affinity of N protein and ssRNA decreases with the addition of 1 μM (**c**), 5 μM (**d**) and 10 μM (**e**) of Ubl1. No interaction between Ubl1 and ssRNA was detected (**c**–**e**, right). The error bar represents the standard error of the mean of three independent experiments (*n* = 3).

The SARS-CoV-2 NTD has been identified to possess an overlapping region between RNA- and Ubl1-binding site (Supplementary Fig. 4), suggesting that Ubl1 and RNA could affect each other for N protein binding. To verify this, we first performed an electrophoretic mobility shift assay (EMSA). The 17-mer transcriptional regulatory sequence (TRS) ssRNA 5′-UGUUCUCUAAACGAACU-3′ (the conserved core sequence of TRS is underlined) in SARS-CoV-2 and its corresponding ssDNA were used. As shown in Fig. 6a, the amount of free ssRNA/ssDNA from the N-RNA mixture increased with the increasing amounts of Ubl1. Ubl1 itself cannot bind to this 17-mer ssRNA/ssDNA (Fig. 6a, lane U); therefore, the increase in free ssRNA/ssDNA indicates that Ubl1 could compete with the binding of N protein to nucleic acids. Additionally, Ubl1 inhibited N-RNA binding in a dose-dependent manner. Subsequently, we quantitatively determined the binding affinity of SARS-CoV-2 N and the 11-mer Cy5-fluorolabeled TRS ssRNA (5′-Cy5-UCUAAACGAAC-3′) using a microscale thermophoresis (MST) assay. The *Kd* was about 5.1 ± 0.6 μM between the N and 11-mer RNA (Fig. 6b). When Ubl1 protein was added to the system, the *Kd* values of N-RNA decreased significantly (Fig. 6c–e). To exclude the possibility of Ubl1 binding to this RNA probe, we investigated the binding affinity between Ubl1 and this 11-mer RNA, and again found no interaction between Ubl1 and RNA (Fig. 6c–e). Therefore, decreased N-RNA binding affinity was induced by Ubl1 competitively binding the N protein. Taken together, these results indicate that SARS-CoV-2 Ubl1 alone does not bind to its TRS RNA. Ubl1 competes with RNA to bind N protein in a dose-dependent manner.

## Discussion

The emerging SARS-CoV-2 and its variants have caused severe damage to the healthcare system globally. Antiviral drugs against SARS-CoV-2 can alleviate the burden of this system. Remdesivir^3^ (Gilead), Paxlovid^4^ (nirmatrelvir and ritonavir mixture, Pfizer), and Molnupiravir^5^ (Merck & Ridgeback) are authorized by the FDA to treat COVID-19 so far. However, the antiviral effects of remdesivir are not fully demonstrated^39,40^, the antiviral resistance of nirmatrelvir has been identified^41,42^, and the host mutagenic risk of molnupiravir is reported^43^. Therefore, the identification of new antiviral targets and the development of novel antiviral drugs are still urgently required. Coronavirus N-Nsp3 interaction plays a crucial role in the viral life cycle^22–25,30^. Understanding the molecular basis of this process is very likely to provide new targets for antiviral therapies.

In 2020, Cong et al. reported that the MHV N protein interacts exclusively with Nsp3 in the RTC. NTD and LKR of the N protein are involved in binding Nps3^N^ (residues 1–233 of Nsp3)^24^. Recently, Koetzner et al. reported that the MHV SR region (within the LKR) directly binds to Ubl1 (residues 1–116) of Nsp3^25^. While Bessa et al. found that the SARS-CoV-2 LKR without SR region binds to Ubl1^33^. In the present study, we narrowed down the N-interacting region of SARS-CoV-2 Nsp3 to residues 1–168 and found that this region independently binds to the N protein. Next, we confirmed that both NTD and LKR are involved in binding to Ubl1 (residues 1–111) of Nsp3. The NTD plus LKR region displayed a stronger Ubl1-binding affinity than the NTD alone, suggesting that the NTD and LKR may cooperatively bind to their partner Ubl1.

**Fig. 7:**
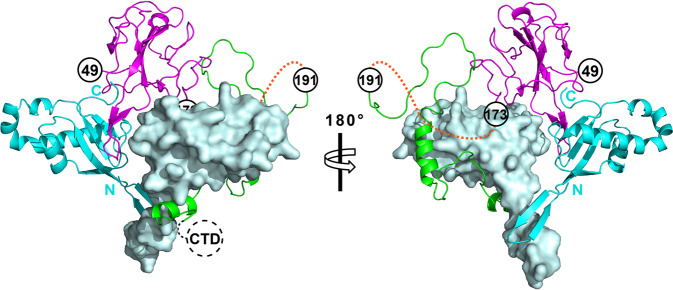
A proposed model for NTD–LKR in complex with Ubl1. Superimposing the previously published NMR structure (PDB code: 7PKU) to our crystal structure (PDB code: 7WZO) enables the illustration of a hypothetical model of NTD–LKR plus Ubl1 complex. The NTD, LKR, chain-B Ubl1, and chain-C Ubl1 are colored in magenta, green, cyan, and light blue, respectively. The N- and C- termini of chain-B Ubl1 are indicated. Chain-C Ubl1 is displayed as the surface view. The SR region (orange dashed lines) is still missing in this model. The NTD interacts with the side near the β-sheets in two Ubl1s, while the LKR binds to the side of the α-helices of Ubl1 (chain C). The CTD following the LKR is indicated by black dashed circles. Figures were prepared using PyMOL (https://pymol.org).

In order to illustrate the relationship between NTD–LKR and Ubl1, the structure of this complex was required. Recently, Bessa et al. reported the NMR structure of the SARS-CoV-2 N-LKR region (denoted N3 in their manuscript) with sUbl1 (residues 16–111) of Nsp3^33^. They found that the LKR is located around sUbl1 through two separate linear motifs (Supplementary Fig. 3). In this study, we identified that NTD and Ubl1 (residues 1–111) could form heterotrimers in the molar ratio of 1:2. The corresponding crystal structure of the NTD-Ubl1 complex was determined. We proposed a model of NTD plus LKR in complex with Ubl1 through superimposing the NMR structure^33^ onto our crystal structure (Fig. 7). According to this model, the NTD mainly interacts with the surfaces near the β-sheets of the two Ubl1s, while the LKR interacts with Ubl1 (chain C) at the side of the α-helices in Ubl1 (Fig. 7), illustrating how NTD and LKR cooperatively bind to Ubl1.

We identified that SARS-CoV-2 Ubl1 exits as a monomer in the SEC-MALS assay, on the other hand, we captured the dominant dimeric Ubl1 in the cross-linking assay. Recently, two reported crystal structures of free SARS-CoV-2 Ubl1 (PDB codes: 7KAG and 7TI9) present an “open V-like” and a “closed V-like” dimer forming by the symmetry-related Ubl1s in the crystal lattice (Supplementary Fig. 5a–c). These two conformations also exist in our NTD-Ubl1 complex structure (Supplementary Fig. 5d). Serrano et al. previously observed the dimeric Ubl1 of SARS-CoV at the protein concentration 1 mM^35^. Wolff et al. found that hexametric Nsp3 located at a small pore in MHV-infection cells^30^, which (very likely) makes Nsp3 to be a transient high local concentration in cells. Therefore, the Ubl1 dimer probably exists during the coronavirus life cycle. It is worth investigating the oligomerization status of Ubl1 (or intact Nsp3) in vivo.

The N protein exists as a dimer. We found that the binding stoichiometry (n) between intact N and Ubl1 is approximately 1 in the ITC assay, indicating a molar ratio of 1 to 1 between these two proteins. However, our crystal structure has already shown that one N protein (the NTD region) interacts with two Ubl1s. Thus, it appears that only one protein of the N dimer binds to Ubl1. What is the possible reason? In the previous studies, Chang et al. revealed that two NTD monomers adopt asymmetric conformations in the SARS-CoV N dimer by SAXS assay^44^. Similarly, Gui et al. observed the asymmetric conformations for NTD monomers in MHV through the electron microscopy method^45^. Most recently, Bessa et al. also showed that the NTD–LKR region (denoted N23 in their manuscript) displays various conformations in the N234 (NTD–LKR–CTD) dimer by performing SAXS experiments^33^. Particularly, NTD–LKR showed obvious conformational compaction when the Ubl1 binding to N234 dimer^33^. Therefore, we suspected that one protein of the N dimer interacting with Ubl1s could cause a conformational change of the NTD–LKR in the other, thus leading to the loss of Ubl1-binding ability in the unbound N protein. To verify this hypothesis, the structure of N in complex with Ubl1 needs to be illustrated in the future.

**Fig. 8:**
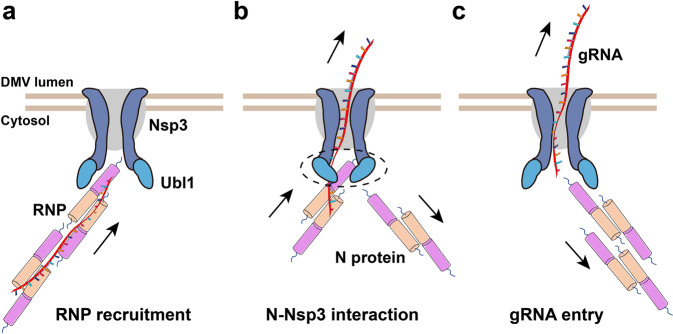
A hypothetical model of viral RNP dissociation through N interacting with Ubl1. **a** Original viral RNP is recruited to the molecular pore located on the DMV surface by N binding to Ubl1 of Nsp3. **b** Two Ubl1 molecules of adjacent Nsp3s interact with N protein to disrupt the N-RNA binding. N protein successively dissociates from the RNP through the dynamic N-Nsp3 (Ubl1) interactions, leading to the delivery of gRNA into DMV. **c** Intact gRNA enters the DMV and serves as a template for viral RNA replication. Free N proteins are released into the cytosol. RNP ribonucleoprotein, DMV double-membrane vesicle, gRNA genomic RNA.

Wolff et al. found that Nsp3 forms the core of a molecular pore with a hexameric structure spanning the bilayer membrane of the DMV^30^. The Ubl1 domain of Nsp3 is located on the cytoplasmic side. They reported that this molecular pore is crucial for viral RNA transport^30^. However, the precise mode by which the molecular pore operates and the mechanism of interaction between N, Nsp3, and RNA around the DMV still require elucidation. Based on our observations, we proposed a model to explain how N-Nsp3 and N-RNA work together to accomplish original viral RNP dissociation and template genomic RNA (gRNA) transportation into the DMV (Fig. 8). First, Nsp3 binds to N protein through its Ubl1 domain, to recruit the original viral RNP to the vicinity of the molecular pore on the DMV surface (Fig. 8a). Then, the Ubl1 domain (high local concentration) of two adjacent Nsp3s completes with RNA to interact with the N protein, leading to the dissociation of RNA and N (Fig. 8b). N protein successively dissociates from the RNP through the dynamic N-Nsp3 (Ubl1) interaction. Finally, the intact gRNA imports into the DMV through the molecular pore and serves as a template for subsequent RNA replication (Fig. 8c). In this proposed model, the hexameric Nsp3 may be divided into three pairs, which could act synergistically to increase the efficiency of the RNP dissociation. More structure information (such as intact N-Nsp3 complex, even including the DMV) needs to be obtained to demonstrate or improve this hypothetical model.

In conclusion, we identified that the N-Nsp3 interaction is preserved in SARS-CoV, MERS-CoV, and SARS-CoV-2 in vitro. We determined the crystal structure of the SARS-CoV-2 N-NTD in complex with Ubl1 of Nsp3 and proved that the NTD-Ubl1 heterotrimer likely exists in solution. We found that Ubl1 competes with RNA to interact with N protein in a dose-dependent manner. By combining our data with the recently published NMR structure^33^, we illustrated that NTD and LKR could cooperatively interact with Ubl1. Finally, we proposed a possible model for viral RNP dissociation through the N-Nsp3 interaction. Overall, our findings expand the knowledge of the interaction between N protein and Nsp3 in coronavirus. The structure of the NTD-Ubl1 complex may provide a potential antiviral target against SARS-CoV-2.

## Methods

### Plasmid constructs

In the fluorescence assay, the SARS-CoV-2 (Genbank: NC_045512.2) *N* gene was amplified via polymerase chain reaction (PCR) using the plasmid *pET28a-N* (GENERAL BIOL. Anhui, China) as the template. The obtained PCR product of the *N* gene was cloned into the *pcDNA3.0-HA-EGFP* vector (HuaYueYang Biotech Co., Ltd., Beijing, China). The full-length *Nsp3* gene was divided into three fragments (*Nsp3.1*: residues 1–654; *Nsp3.2*: 649–1304; and *Nsp3.3*: 1299–1945). Three fragments were amplified using the plasmid *pFast-Nsp3* (gift from the Protein Bank of National Facility for Protein Science, Shanghai, China) as the template. The *Nsp3.3* was cloned into the *pCMV-mCherry* vector (Beyotime, Shanghai, China) between the SacI and EcoRI sites. The *Nsp3.2* and *Nsp3.1* were sequentially inserted into the forward site of *Nsp3.3* using Hieff Clone^®^ Plus One Step Cloning Kit (Yeasen, Shanghai, China), to obtain *pCMV-Nsp3-mCherry* plasmid. *pCMV-Nsp3*^*Δ111*^*-mCherry* (*Nsp3*^*Δ111*^: deleted the N-terminal 111 residues) and *pCMV-Nsp3*^*Δ168*^*-mCherry* plasmids were obtained through the similar method. *Ubl1* (residues 1–111 of Nsp3) fragment was amplified from the *pFast-Nsp3* vector and directly cloned into the *pCMV-mCherry* vector using the Hieff Clone^®^ Plus One Step Cloning Kit. Primers used for PCR are listed in Supplementary Table 1. The recombinant plasmid DNA was verified by sequencing (Tsingke Biotechnology Co., Ltd., Chengdu, China).

In the rest of the assays, constructs of SARS-CoV-2 *N* (residues 1–419), *N* truncations (1–247, 47–174, 47–247, and 248–419), and *Ubiquitin-like domain 1 (Ubl1)* of *Nsp3* (residues 1–111) were amplified by PCR using the above-mentioned plasmids as the templates. All primers are shown in Supplementary Table 2. The *N* and *N* truncations were ligated into the modified *pET28a* plasmids, containing an N-terminal hexahistidine tag and a Tobacco Etch Virus protease (TEV) cleavage site. While the *Ubl1* of the *Nsp3* construct was cloned into the unmodified *pET28a* plasmids (Novagen). Single-point mutations (R92A in N-NTD, *E26A*, *E95A*, *Y103A*, and *D110A* in Ubl1) were performed by site-directed mutagenesis. The corresponding primers used for these mutations are listed in Supplementary Table 3. All recombinant plasmids were identified by sequencing (Tsingke Biotechnology Co., Ltd., Chengdu, China).

The plasmids *pET28a*-*N* and *pET28a*-*Ubl1* (containing the homologous genes *N* and *Ubl1* in SARS-CoV (GenBank: NC_004718.3) and MERS-CoV (GenBank: NC_019843.3)) were purchased from GENERAL BIOL., Anhui, China.

### Cell culture and transfection

Huh-7 cells (Thermo Fisher) were grown in Dulbecco’s modified Eagle’s medium (DMEM; BI) with 10% (v/v) fetal bovine serum (Gibco, USA), 100 U/mL penicillin, and 100 μg/mL streptomycin in a humidified incubator at 37 °C and 5% CO_2_. For the colocalization assay, Huh-7 cells were seeded directly onto precision coverslips and cultured in 24-well plates. Co-transfection of recombinant *pCMV-Nsp3-mCherry* and *pcDNA3.0-N-HA-EGFP* plasmids were carried out with Transfection reagent PEI (Thermo Fisher) according to the manufacturer’s protocol when the cells were 70% confluent. Control groups were performed with the same approach. After 48 h post-transfection, cells were fixed with 4% paraformaldehyde for 10 min and then washed three times with PBST buffer. Then cells were stained with DAPI to show the cell nucleus. Pictures were subsequently taken with an Olympus Spin co-focus fluorescence microscope (Olympus Life Science).

### Gene expression and protein purification

All plasmids for in vitro assay were expressed in the same way. No gene name is specified for the procedure of gene expression here. The corrected recombinant plasmids were transformed into *E. coli* BL21(DE3) (Novagen). Then the cells were grown overnight at 37 °C in 50 mL Luria-Broth (LB) medium supplemented with kanamycin at a final concentration of 50 μg/mL. The culture was next inoculated into 2 × 1 L LB medium on the following day. When the OD_600_ value of the culture reached 0.6–0.8, overexpression of the target gene was induced for 16 h with 0.5 mM isopropyl-β-d-thiogalactoside (IPTG) supplementation at 18 °C. The 2 L culture was subsequently harvested by centrifugation for 15 min, 4000 rpm at 4 °C. Then the harvested *E. coli* BL21(DE3) cells were resuspended in 50 mL buffer A (20 mM Tris-HCl, 10 mM imidazole, 1 M NaCl, pH 7.5) and lysed by ultrasonication on ice. After centrifugation for 30 min at 18,000 rpm at 4 °C, the supernatant of the lysed mixture was applied to Ni Sepharose™ Fast Flow beads (GE Healthcare). Next, the His-tagged protein was eluted by buffer B (20 mM Tris-HCl, 500 mM imidazole, 500 mM NaCl, pH 7.5) with a step-gradient method. Then, the His-tag of the target protein was cleaved by TEV protease (prepared by ourselves) for N and N-truncated proteins or thrombin (Sigma) for Ubl1. The corresponding protein was subsequently dialyzed against buffer C (20 mM Tris-HCl, 150 mM NaCl, 5% Glycerol, pH 7.5) overnight at 4 °C. After His-tag removal, the N protein (and/or the N-truncated proteins) in SARS-CoV-2, SARS-CoV and MERS-CoV were further applied to Heparin column (GE Healthcare) to remove the unspecific interacting nucleic acids. On the other hand, the Ubl1s in three CoVs were applied to the Q column (GE Healthcare) for further purification. Finally, all target proteins were purified again by size-exclusion chromatography (SEC) using buffer C, and the quality of these proteins was identified by SDS-PAGE.

### Crystallization and data collection

Purified NTD of the SARS-CoV-2 N protein was mixed with Ubl1 at a molar ratio of 1:2, and the mixture was incubated at 4 °C overnight. This mixture was purified by SEC (Superdex 75 Increase 10/300 GL, GE Healthcare) the next day. Both NTD and NTD-Ubl1 complex were concentrated to ~40 mg/mL in buffer C. The crystallization trials for these two samples were performed at 291 K by employing the sitting-drop vapor-diffusion method with 1 µL of protein plus 1 µL of the reservoir. Crystals of NTD were observed under the condition of No. 28 of the kit Crystal screen^TM^ (Hampton research): 0.2 M sodium acetate trihydrate, 0.1 M sodium cacodylate pH 6.5, 30% w/v PEG 8000. The optimized crystals were subsequently produced under the conditions: 0.2 M sodium acetate trihydrate, 0.1 M sodium cacodylate pH 7.5, 34% w/v PEG 8000. While crystals of the NTD-Ubl1 complex were obtained under the condition of No. E11 of the kit ProPlex^TM^ HT-96 (Molecular Dimensions): 0.1 M sodium citrate, pH 5.0, 20% w/v PEG 8000. The NTD-Ubl1 complex crystals were further dehydrated to improve the X-ray diffraction power. All the above crystals were shock-cooled in liquid nitrogen. The diffraction data sets of NTD (~1.95 Å) and NTD-Ubl1 (~2.64 Å) were collected with an X-ray wavelength of 0.97581 Å at the Shanghai synchrotron radiation facility (SSRF) beamline BL19U1, Shanghai, China. Two data sets were processed by *XDS*^46^ and scaled with *Aimless* in *CCP4*^47^. The space group of NTD crystal is $P{\it{2}_{1}}{\it{2}_{1}}{\it{2}_{1}}$ with unit-cell parameters: a = 58.71 Å, b = 91.41 Å, c = 96.12 Å, α = β = γ = 90°. While the space group of the NTD-Ubl1 complex is *P3*_*1*_*21* with unit-cell parameters: a = b = 69.54 Å, c = 269.25 Å, γ = 120°. The detailed crystallographic statistics are presented in Table 2.

### Phase determination and model refinement

Both structures of the SARS-CoV-2 N-NTD and NTD-Ubl1 complex were determined by the molecular replacement (MR) method. The initial phase of SARS-CoV-2 N-NTD was determined by MR with program *MOLREP*^48^, using the SARS-CoV N-NTD^49^ (PDB code: 2OFZ) as the search model. Next, the initial model of the SARS-CoV-2 N-NTD was rebuilt and refined using programs *Coot*^50^ and *REFMAC5*^51^. The final *R*_factor_ is 0.188, with *R*_free_ = 0.226. Subsequently, the structure of the SARS-CoV-2 N-NTD in complex with Ubl1 was determined with program *MOLREP*^48^, using our N-NTD (PDB code: 7VNU) as the first search model. Then, we fixed the initial N-NTD model, and performed the program *MOLREP*^48^ again using the Ubl1 monomer (PDB code: 7KAG) as the second search model. Afterwards, we obtained the corrected initial phase of NTD in a complex with Ubl1. The primary NTD-Ubl1 model was continually rebuilt and refined using programs *Coot*^50^ and *PHENIX. refine*^52^. The *R*_factor_ and *R*_free_ of the NTD-Ubl1 complex are 0.214 and 0.246, respectively. The *Ramachandran plot* statistics of NTD and NTD-Ubl1 complex structures present that 98.0 and 96.0% of the residues lie within the preferred region, respectively, 2.0 and 4.0% of the residues within the allowed region, respectively, and no residues within the outlier region. Please see Table 2 for the final refinement statistics.

### Isothermal titration calorimetry (ITC) assay

Freshly purified SARS-CoV-2 N and SARS-CoV N were concentrated to 20 μM in ITC buffer (20 mM HEPES, 150 mM NaCl, pH 7.5). Their cognate Ubl1s were adjusted to 300 μM in the same buffer. In parallel, MERS-CoV N was concentrated to 75 μM and its cognate Ubl1 was adjusted to 1 mM. The measurements of binding affinity were carried out using a Nano ITC (Nano ITC-Low Volume; TA instrument, USA). The titration assay was performed at 16 °C by injecting 2.5 μL Ubl1 into a full cell containing 190 μL cognate N every 120 s for a total of 20 injections, and the stirrer syringe stirring speed was set at 250 rpm.

To measure the binding affinity between SARS-CoV-2 Ubl1 and various truncations (N-arm–NTD–LKR, NTD–LKR, NTD, and CTD–C-tail) of cognate N protein, N protein truncation was used as a ligand-protein in the injection syringe to obtain better fitting curves. The experimental concentrations of the different SARS-CoV-2 N truncations were adjusted from 300 μM to 1 mM, and the corresponding Ubl1 concentrations were adjusted from 50 to 100 μM. In the ITC assay between NTD R92A and Ubl1 or NTD and Ubl1 mutations (*E26A*, *E95A*, *Y103A*, or *D110A*), the working concentration of each mutation was adjusted to around the corresponding concentration of the wild type. All raw titration datasets were processed in NanoAnalyze Data Analysis software, version 3.8.0.

### SEC-MALS assay

To directly measure the molecular mass of SARS-CoV-2 Ubl1 in solution, the size-exclusion chromatography coupled to multi-angle light scattering (SEC-MALS) assay was performed. The purified 50 μL Ubl1 (~5 mg/mL) protein was injected into column WTC-030S5 (Wyatt Technology, USA) with a flow rate of 0.75 mL/min. The light scattering signal and the refractive index profile were collected with the DAWN and Optilab (Wyatt Technology, USA). The molecular mass was calculated by the software ASTRA 8 (Wyatt Technology, USA).

### Cross-linking assays of SARS-CoV-2 Ubl1 and NTD-Ubl1 complex

The purified Ubl1 (~10 μg) and NTD-Ubl1 (~10 μg) complex were dissolved in 10 μL cross-linking buffer (20 mM HEPES, 150 mM NaCl, pH 7.5) and cross-linked with 0.1% v/v glutaraldehyde (1 μL of 1% v/v) for 5, 10, 20 min at 37 °C, respectively. 10 μL of 1 M Tris-HCl (pH 8.0) was added into each tube to stop the reaction. Subsequently, 7 μL of denaturing sample buffer (4x) was added to the reaction sample, and this mixture was heated at 95 °C for 2 min. Finally, 5 μg of protein was separated by SDS-PAGE to identify the products after the cross-linking reaction.

### Dynamic light scattering (DLS) assay

The freshly purified SARS-CoV-2 NTD-Ubl1 complex was concentrated to 4 mg/mL in DLS buffer (20 mM Tris-HCl, 150 mM NaCl, pH 7.5). This sample in a volume of 10 μL were tested at 25 °C using DynaPro NanoStar (Wyatt Technology, USA) in three independent replicate experiments (*n* = 3). The hydrodynamic radius (R_*h*_) and molecular mass (MW) of the NTD-Ubl1 complex was calculated by the software DYNAMICS (Wyatt Technology, USA). The conversion formula between R_*h*_ and MW is: MW (kD) = [R_*h*_ Factor × R_*h*_ (nm)]^Power^, in which the R_*h*_ Factor and Power are 1.6800 and 2.3398 for proteins (DYNAMICS User’s Guide).

### Electrophoretic mobility shift assay (EMSA)

Both the 17-mer ssRNA (5′-UGUUCUCUAAACGAACU-3′) and 17-mer ssDNA (5′-TGTTCTCTAAACGAACT-3′) oligonucleotides were synthesized by Youkang Biological Technology (Chengdu, China) and further dissolved in EMSA buffer (20 mM HEPES, 150 mM NaCl, pH 7.5). The conserved core sequence of the transcriptional regulatory sequence RNA in SARS-CoV-2 is underlined. The SARS-CoV-2 N protein and Ubl1 were concentrated to a stock concentration of 15 μM in EMSA buffer. Each reaction system contained the same final concentration of oligonucleotide (2.5 μM), and ssRNA (or ssDNA) alone, Ubl1 (3.8 μM) + ssRNA (or ssDNA) and N protein (3.8 μM) + ssRNA (or ssDNA) were included as controls. The oligonucleotide (ssRNA or ssDNA) and N protein were mixed with increasing concentrations of Ubl1, and the molar ratios of N protein to Ubl1 in the different tubes were 5:1, 5:2, 5:3, 5:4, and 5:5. All reaction systems were brought to a volume of 20 μL with EMSA buffer, and then incubated at 4 °C for 10 min. Finally, each sample was separated by 1% agarose gel electrophoresis on the ice at 60 V for 50 min, and the results were visualized using a Tanon-3500B imager (Tanon Co., Ltd., Shanghai, China).

### Microscale thermophoresis (MST) binding assay

Purified SARS-CoV-2 N protein and Ubl1 were diluted in MST buffer (20 mM HEPES, 150 mM NaCl, pH 7.5, and 0.05% Tween-20) at concentrations of 200 and 40 μM, respectively. A fluorophore-labeled 11-mer ssRNA (5′-Cy5-UCUAAACGAAC-3′) was synthesized by the Youkang Biology Company, Chengdu, China. The 11-mer ssRNA was dissolved in MST buffer at a concentration of 160 nM. For the first set in the MST assay, 10 μL of SARS-CoV-2 N protein (concentration range 1.5 nM - 50 μM) was added to 16 tubes with the serial dilution method. Then, 5 μL ssRNA (stock concentration 160 nM) and 5 μL MST buffer were pipetted into each tube. For other sets in the assay, 5 μL MST buffer was replaced with 5 μL of Ubl1 at stock concentrations of 4, 20, and 40 μM, respectively. The ssRNA samples (final concentration 40 nM) mixing with Ubl1 protein (final concentrations 1, 5, 10 μM) were used as the control groups. For each set, each tube was mixed well and incubated for 30 min at 23 °C. Subsequently, all reaction samples were loaded into standard capillaries for measurements at 23 °C, 20% LED-power, and medium MST power using a Monolith NT.115 instrument (NanoTemper Technologies). Data from three independent measurements (*n* = 3) of the signal corresponding to the 5 s MST-On time were analyzed using MO.Affinity Analysis software (NanoTemper Technologies).

### Reporting summary

Further information on research design is available in the Nature Portfolio Reporting Summary linked to this article.

## Supplementary information

Supplementary Information

Reporting Summary

## Data Availability

Atomic coordinates and structure factors for SARS-CoV-2 N-NTD and NTD-Ubl1 complex have been deposited in the Protein Data Bank with the accession codes 7VNU and 7WZO, respectively. Uncropped gels are provided in Supplementary Fig. 6.

## References

[1] Tanriover MD (2021). Efficacy and safety of an inactivated whole-virion SARS-CoV-2 vaccine (CoronaVac): interim results of a double-blind, randomised, placebo-controlled, phase 3 trial in Turkey. Lancet.

[2] Mateus J (2021). Low-dose mRNA-1273 COVID-19 vaccine generates durable memory enhanced by cross-reactive T cells. Science.

[3] Beigel JH (2020). ACTT-1 study group members (2020). Remdesivir for the treatment of covid-19—final report. N. Engl. J. Med..

[4] Owen DR (2021). An oral SARS-CoV-2 M^pro^ inhibitor clinical candidate for the treatment of COVID-19. Science.

[5] Wahl A (2021). SARS-CoV-2 infection is effectively treated and prevented by EIDD-2801. Nature.

[6] Drosten C (2003). Identification of a novel coronavirus in patients with severe acute respiratory syndrome. N. Engl. J. Med..

[7] Zaki AM, van Boheemen S, Bestebroer TM, Osterhaus AD, Fouchier RA (2012). Isolation of a novel coronavirus from a man with pneumonia in Saudi Arabia. N. Engl. J. Med..

[8] Hagemeijer MC (2010). Dynamics of coronavirus replication-transcription complexes. J. Virol..

[9] Chang CK, Hou MH, Chang CF, Hsiao CD, Huang TH (2014). The SARS coronavirus nucleocapsid protein–forms and functions. Antivir. Res..

[10] Bai Z, Cao Y, Liu W, Li J (2021). The SARS-CoV-2 nucleocapsid protein and its role in viral structure, biological functions, and a potential target for drug or vaccine mitigation. Viruses.

[11] Peng Y (2020). Structures of the SARS-CoV-2 nucleocapsid and their perspectives for drug design. EMBO J..

[12] Savastano A, Ibáñez de Opakua A, Rankovic M, Zweckstetter M (2020). Nucleocapsid protein of SARS-CoV-2 phase separates into RNA-rich polymerase-containing condensates. Nat. Commun..

[13] Iserman C (2020). Genomic RNA elements drive phase separation of the SARS-CoV-2 nucleocapsid. Mol. Cell.

[14] Lu S (2021). The SARS-CoV-2 nucleocapsid phosphoprotein forms mutually exclusive condensates with RNA and the membrane-associated M protein. Nat. Commun..

[15] Perdikari TM (2020). SARS-CoV-2 nucleocapsid protein phase-separates with RNA and with human hnRNPs. EMBO J..

[16] Pan P (2021). SARS-CoV-2 N protein promotes NLRP3 inflammasome activation to induce hyperinflammation. Nat. Commun..

[17] Kuo L, Masters PS (2002). Genetic evidence for a structural interaction between the carboxy termini of the membrane and nucleocapsid proteins of mouse hepatitis virus. J. Virol..

[18] van der Meer Y (1999). Localization of mouse hepatitis virus nonstructural proteins and RNA synthesis indicates a role for late endosomes in viral replication. J. Virol..

[19] Sims AC, Ostermann J, Denison MR (2000). Mouse hepatitis virus replicase proteins associate with two distinct populations of intracellular membranes. J. Virol..

[20] Stertz S (2007). The intracellular sites of early replication and budding of SARS-coronavirus. Virology.

[21] Ulasli M, Verheije MH, de Haan CA, Reggiori F (2010). Qualitative and quantitative ultrastructural analysis of the membrane rearrangements induced by coronavirus. Cell Microbiol..

[22] Hurst KR, Ye R, Goebel SJ, Jayaraman P, Masters PS (2010). An interaction between the nucleocapsid protein and a component of the replicase-transcriptase complex is crucial for the infectivity of coronavirus genomic RNA. J. Virol..

[23] Hurst KR, Koetzner CA, Masters PS (2013). Characterization of a critical interaction between the coronavirus nucleocapsid protein and nonstructural protein 3 of the viral replicase-transcriptase complex. J. Virol..

[24] Cong Y (2020). Nucleocapsid protein recruitment to replication-transcription complexes plays a crucial role in coronaviral life cycle. J. Virol..

[25] Koetzner CA, Hurst-Hess KR, Kuo L, Masters PS (2022). Analysis of a crucial interaction between the coronavirus nucleocapsid protein and the major membrane-bound subunit of the viral replicase-transcriptase complex. Virology.

[26] Lei J, Kusov Y, Hilgenfeld R (2018). Nsp3 of coronaviruses: structures and functions of a large multi-domain protein. Antivir. Res..

[27] van Hemert MJ (2008). SARS-coronavirus replication/transcription complexes are membrane-protected and need a host factor for activity in vitro. PLoS Pathog..

[28] Oudshoorn D (2017). Expression and cleavage of middle east respiratory syndrome coronavirus nsp3-4 polyprotein induce the formation of double-membrane vesicles that mimic those associated with coronaviral RNA replication. mBio.

[29] Jiang Y (2021). Genome-wide analysis of protein-protein interactions and involvement of viral proteins in SARS-CoV-2 replication. Cell Biosci..

[30] Wolff G (2020). A molecular pore spans the double membrane of the coronavirus replication organelle. Science.

[31] Dinesh DC (2020). Structural basis of RNA recognition by the SARS-CoV-2 nucleocapsid phosphoprotein. PLoS Pathog..

[32] Luan X (2022). Antiviral drug design based on structural insights into the N-terminal domain and C-terminal domain of the SARS-CoV-2 nucleocapsid protein. Sci. Bull..

[33] Bessa LM (2022). The intrinsically disordered SARS-CoV-2 nucleoprotein in dynamic complex with its viral partner nsp3a. Sci. Adv..

[34] Keane SC, Giedroc DP (2013). Solution structure of mouse hepatitis virus (MHV) nsp3a and determinants of the interaction with MHV nucleocapsid (N) protein. J. Virol..

[35] Serrano P (2007). Nuclear magnetic resonance structure of the N-terminal domain of nonstructural protein 3 from the severe acute respiratory syndrome coronavirus. J. Virol..

[36] Kabsch W, Sander C (1983). Dictionary of protein secondary structure: pattern recognition of hydrogen-bonded and geometrical features. Biopolymers.

[37] Krissinel E, Henrick K (2007). Inference of macromolecular assemblies from crystalline state. J. Mol. Biol..

[38] Salvi N (2021). 1H, 13C and 15N backbone chemical shift assignments of SARS-CoV-2 nsp3a. Biomol. NMR Assign..

[39] Ader F (2022). Remdesivir plus standard of care versus standard of care alone for the treatment of patients admitted to hospital with COVID-19 (DisCoVeRy): a phase 3, randomised, controlled, open-label trial. Lancet Infect. Dis..

[40] Pagliano P (2022). An overview of the preclinical discovery and development of remdesivir for the treatment of coronavirus disease 2019 (COVID-19). Expert Opin. Drug Discov..

[41] Zhou Y (2022). Nirmatrelvir-resistant SARS-CoV-2 variants with high fitness in an infectious cell culture system. Sci. Adv..

[42] Jochmans, D. et al. The substitutions L50F, E166A, and L167F in SARS-CoV-2 3CLpro are selected by a protease inhibitor in vitro and confer resistance to nirmatrelvir. *mBio* e0281522 (2023). 10.1128/mbio.02815-22 PMC9973015 36625640

[43] Zhou S (2021). β-_D_-*N*^4^-hydroxycytidine inhibits SARS-CoV-2 through lethal mutagenesis but is also mutagenic to mammalian cells. J. Infect. Dis..

[44] Chang CK (2009). Multiple nucleic acid binding sites and intrinsic disorder of severe acute respiratory syndrome coronavirus nucleocapsid protein: implications for ribonucleocapsid protein packaging. J. Virol..

[45] Gui M (2017). Electron microscopy studies of the coronavirus ribonucleoprotein complex. Protein Cell.

[46] Kabsch W (2010). XDS. Acta Crystallogr. D. Biol. Crystallogr..

[47] Evans PR, Murshudov GN (2013). How good are my data and what is the resolution?. Acta Crystallogr. D. Biol. Crystallogr..

[48] Vagin A, Teplyakov A (2009). Molecular replacement with MOLREP. Acta Crystallogr. D. Biol. Crystallogr..

[49] Saikatendu KS (2007). Ribonucleocapsid formation of severe acute respiratory syndrome coronavirus through molecular action of the N-terminal domain of N protein. J. Virol..

[50] Emsley P, Lohkamp B, Scott WG, Cowtan K (2010). Features and development of Coot. Acta Crystallogr. D. Biol. Crystallogr..

[51] Kovalevskiy O, Nicholls RA, Long F, Carlon A, Murshudov GN (2018). Overview of refinement procedures within REFMAC5: utilizing data from different sources. Acta Crystallogr. D. Biol. Crystallogr..

[52] Adams PD (2010). PHENIX: a comprehensive Python-based system for macromolecular structure solution. Acta Crystallogr. D. Biol. Crystallogr..

