# Supplementary material for: Structural insights into ribonucleoprotein dissociation by nucleocapsid protein interacting with non-structural protein 3 in SARS-CoV-2
Source: Commun Biol. 2023 Feb 18;6:193. doi: 10.1038/s42003-023-04570-2 (PMC9938351; doi:10.1038/s42003-023-04570-2)
Supplement: Supplementary file 1 — Supplementary Information [file 42003_2023_4570_MOESM1_ESM.pdf]

Supplementary Information for

Structural insights into ribonucleoprotein dissociation by nucleocapsid protein interacting with non-structural protein 3 in SARS-CoV-2

Xincheng Ni, Yinze Han, Renjie Zhou, Yanmei Zhou, Jian Lei*

*Corresponding author. Email: leijian@scu.edu.cn

This file includes:

Supplementary Figures 1–6

Supplementary Tables 1–3

References 1–3

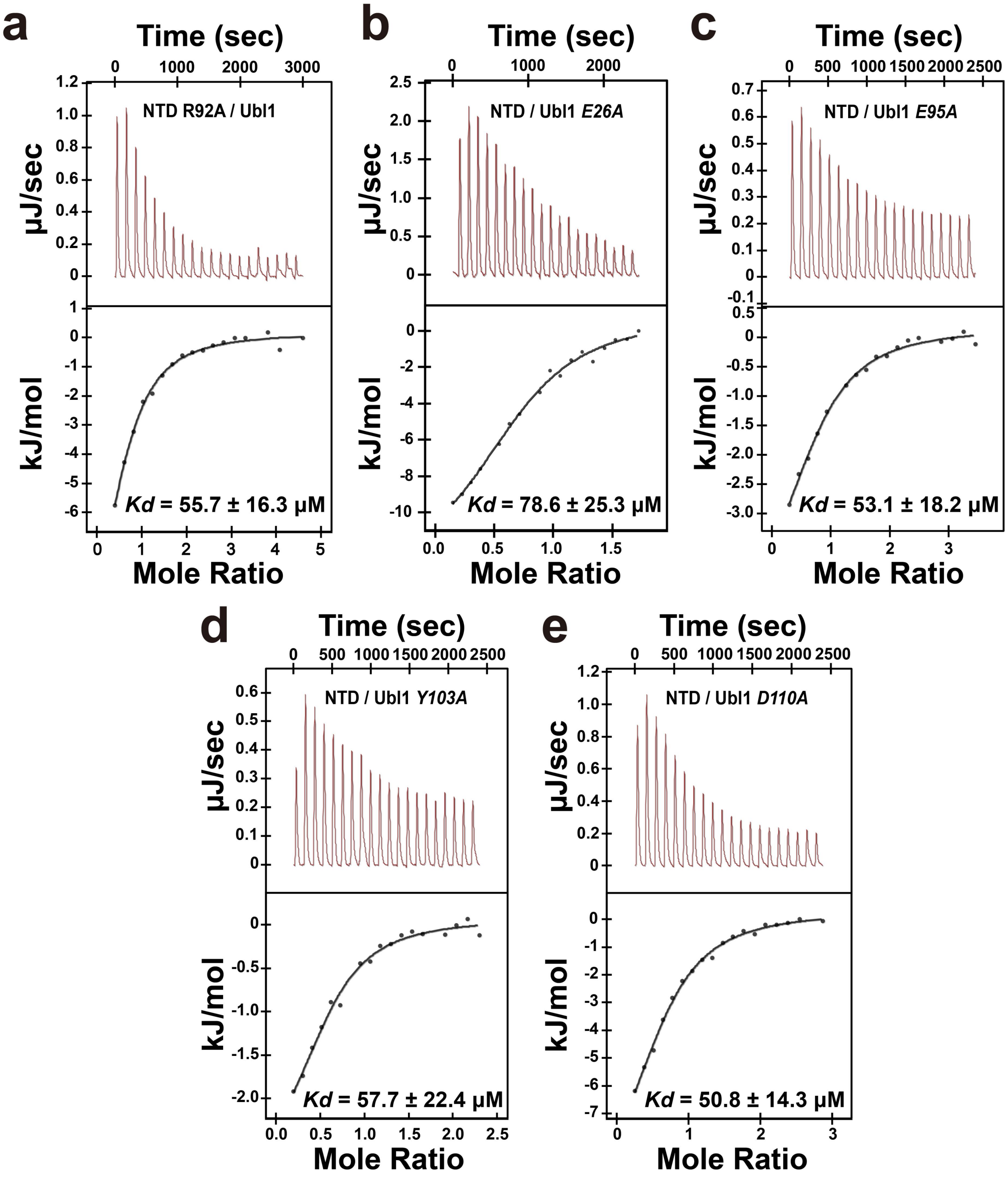

Supplementary Fig. 1. Affinity measurements of SARS-CoV-2 NTD and Ubl1 mutations. **a** Affinity between NTD R92A and Ubl1. **b-e** Affinity between NTD and Ubl1 *E26A* (**b**), *E95A* (**c**), *Y103A* (**d**), and *D110A* (**e**). The raw calorimetric curve is shown in the top panel, while the fitted binding isotherm curve is displayed in the bottom panel. *Kd*: dissociation constant.

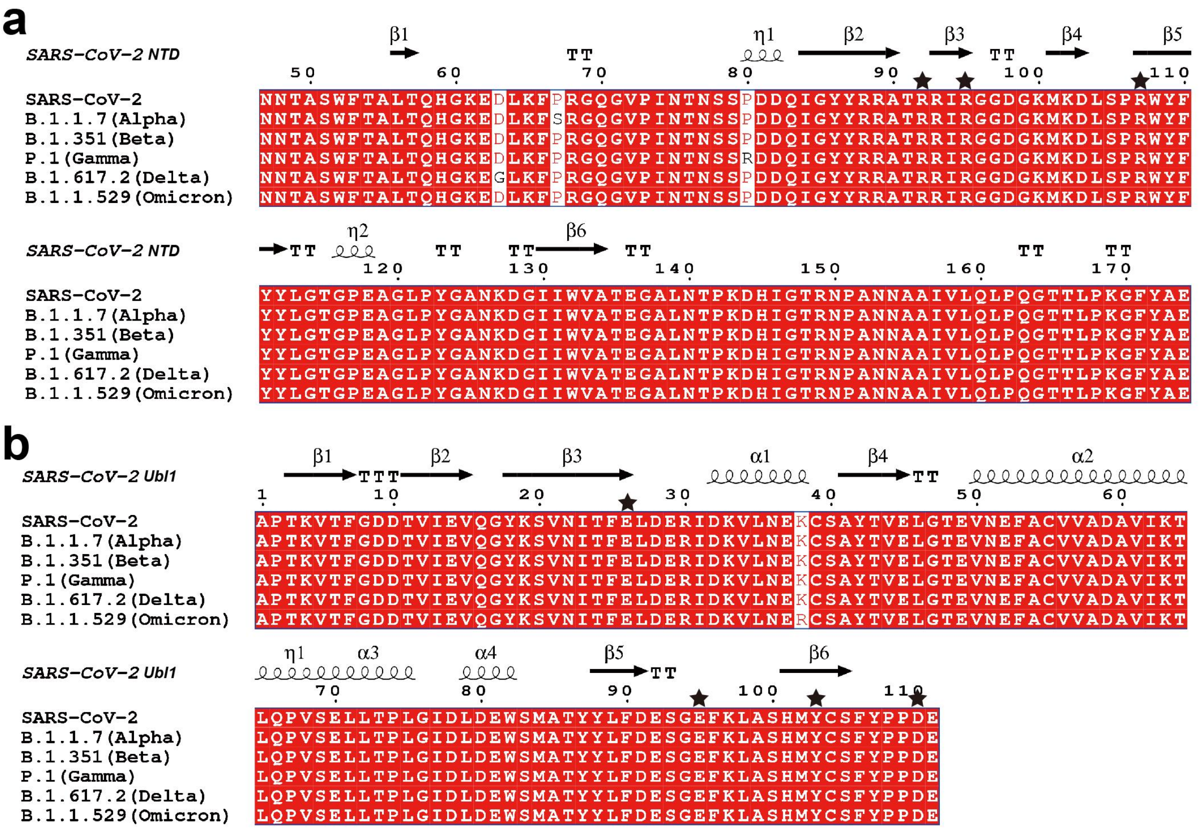

Supplementary Fig. 2. Structure-based multiple sequence alignment of NTD (a) and Ubl1 (b) in SARS-CoV-2 and its variant strains. The corresponding sequence accession numbers are: SARS-CoV-2, GenBank: NC_045512.2; B.1.1.7, GenBank: MZ622698; B.1.351, GenBank: MZ433432; P.1, GenBank: MZ477759; B.1.617.2, GenBank: OK091006; B.1.1.529, GenBank: OL672836. The secondary structures of SARS-CoV-2 NTD (PDB code: 7VNU) and Ubl1 (chain B) in NTD-Ubl1 complex (PDB code: 7WZO) are indicated. Residues R92, R95, R107 of NTD and *E26*, *E95*, *Y103*, *D110* of Ubl1 are labeled with black stars. Figures (**a** and **b**) were prepared by the program ESPript[1].

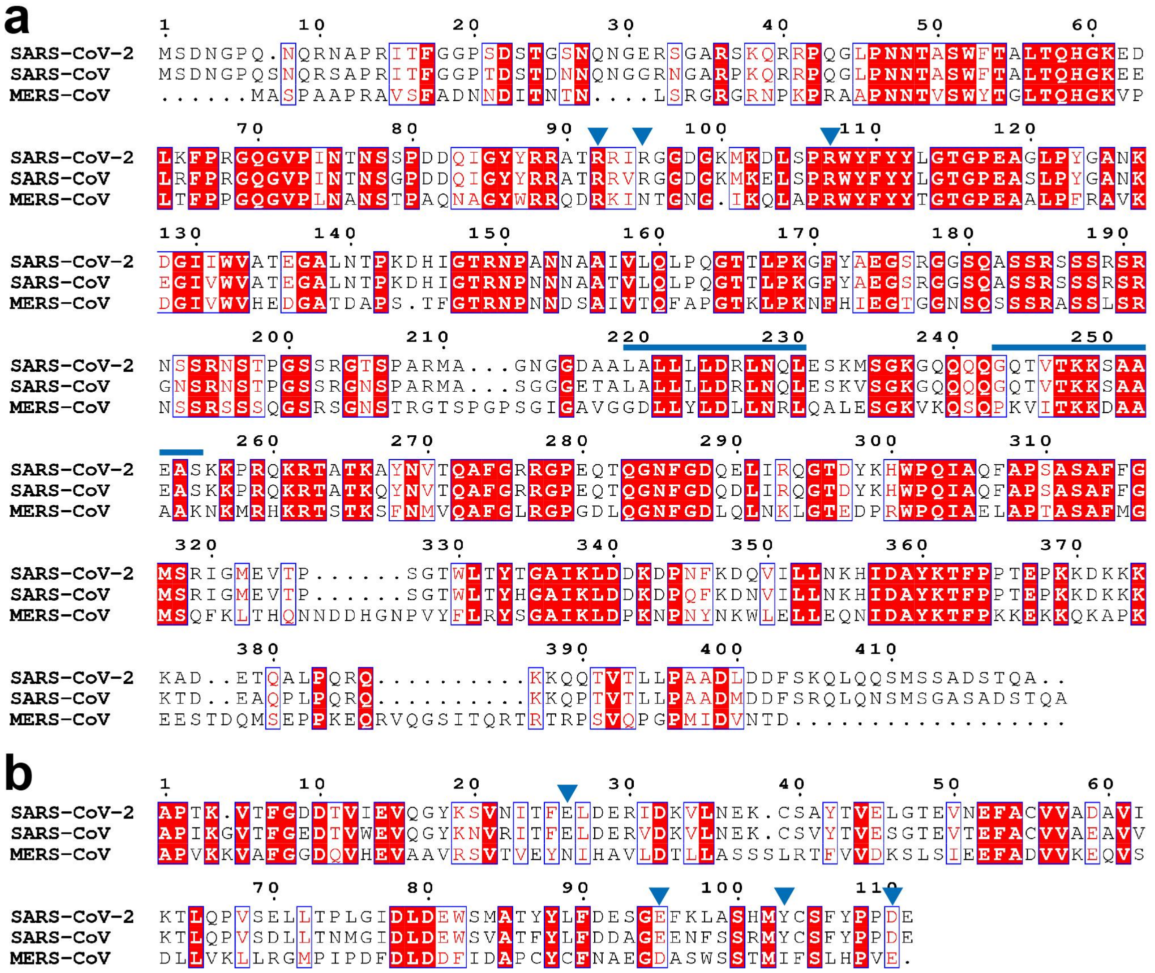

Supplementary Fig. 3. Multiple sequence alignment of N protein (a) and Ubl1 (b) among SASRS-CoV-2, SARS-CoV and MERS-CoV. The sequence accession numbers are: SARS-CoV-2, GenBank: NC_045512.2; SARS-CoV, GenBank: NC_004718.3; MERS-CoV, GenBank: NC_019843.3. Key residues for SARS-CoV-2 NTD-Ubl1 interaction are marked by blue-triangle arrows. Two Ubl1-binding motifs[2] (^{219}LALLLLDRLNQL^{230} and ^{243}GQTVTKKSAAEAS^{255}) of SARS-CoV-2 N-LKR are indicated by the blue lines. Figures (**a** and **b**) were generated using the program ESPript[1].

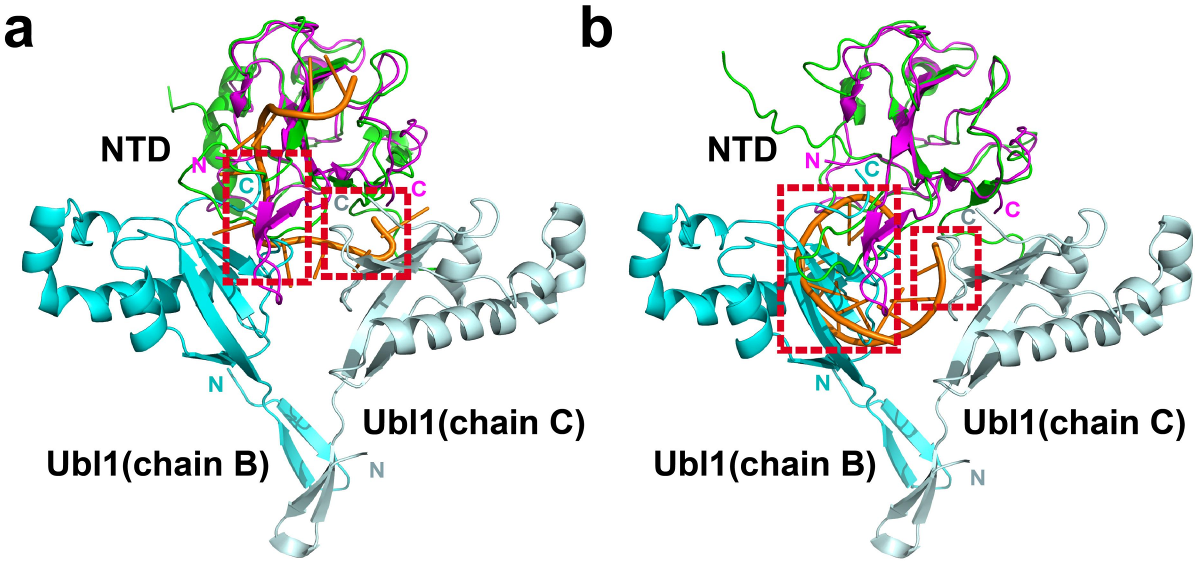

Supplementary Fig. 4. Overlapping region between Ubl1- and (ss/ds) RNA- binding site of SARS-CoV-2 N protein. a Superimposing the NTD-ssRNA complex structure[3] (PDB code: 7ACT) to our NTD-Ubl1 complex structure (PDB code: 7WZO). **b** Superimposing the NTD-dsRNA complex structure[3] (PDB code: 7ACS) to our crystal structure. NTD of the NTD-Ubl1 complex is shown in magenta. While NTD in the NTD-(ds/ss) RNA complex is displayed in green. Chain-B Ubl1 and chain-C Ubl1 are colored in cyan and light blue, respectively. The N- and C- termini of NTD and Ubl1s are labeled in the corresponding colors. Both the ssRNA and dsRNA are colored in orange. The overlapping regions between Ubl1s and (ss/ds) RNA are indicated by red dashed boxes. Figures (**a** and **b**) were prepared using PyMOL (https://pymol.org).

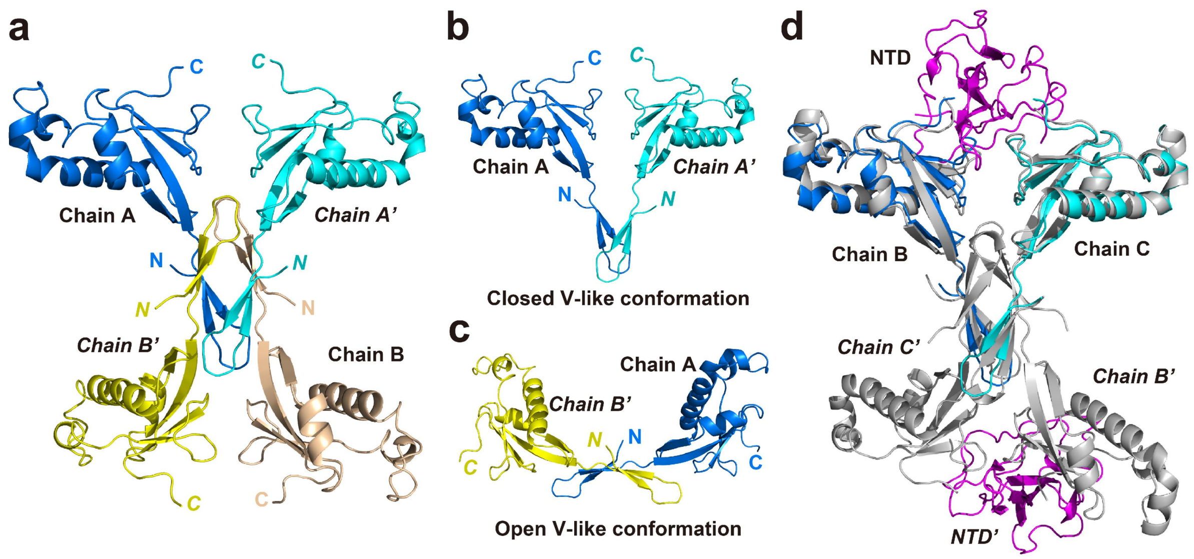

Supplementary Fig. 5. Two different conformations of the potential dimeric Ubl1 in the crystal structure. a A “closed V-like” (Chain A and *A′*) and an “open V-like” (Chain A and *B′*) conformations forming by the symmetry-related Ubl1s in the crystal structure of SARS-CoV-2 Ubl1 (PDB code: 7KAG). Ubl1 chain A, chain B, and their symmetrical protomers are colored in blue, brown, cyan, and yellow, respectively. **b** The “closed V-like” conformation of dimeric Ubl1. **c** The “open V-like” conformation of dimeric Ubl1. **d** Superimposing the dimeric “closed V-like” Ubl1 structure to our NTD-Ubl1 complex structure (PDB code: 7WZO). The Ubl1s in the NTD-Ubl1 complex and their symmetrical molecules are colored in gray. The NTD and its symmetrical protomer are displayed in magenta. Figures (**a-d**) were prepared using PyMOL (https://pymol.org).

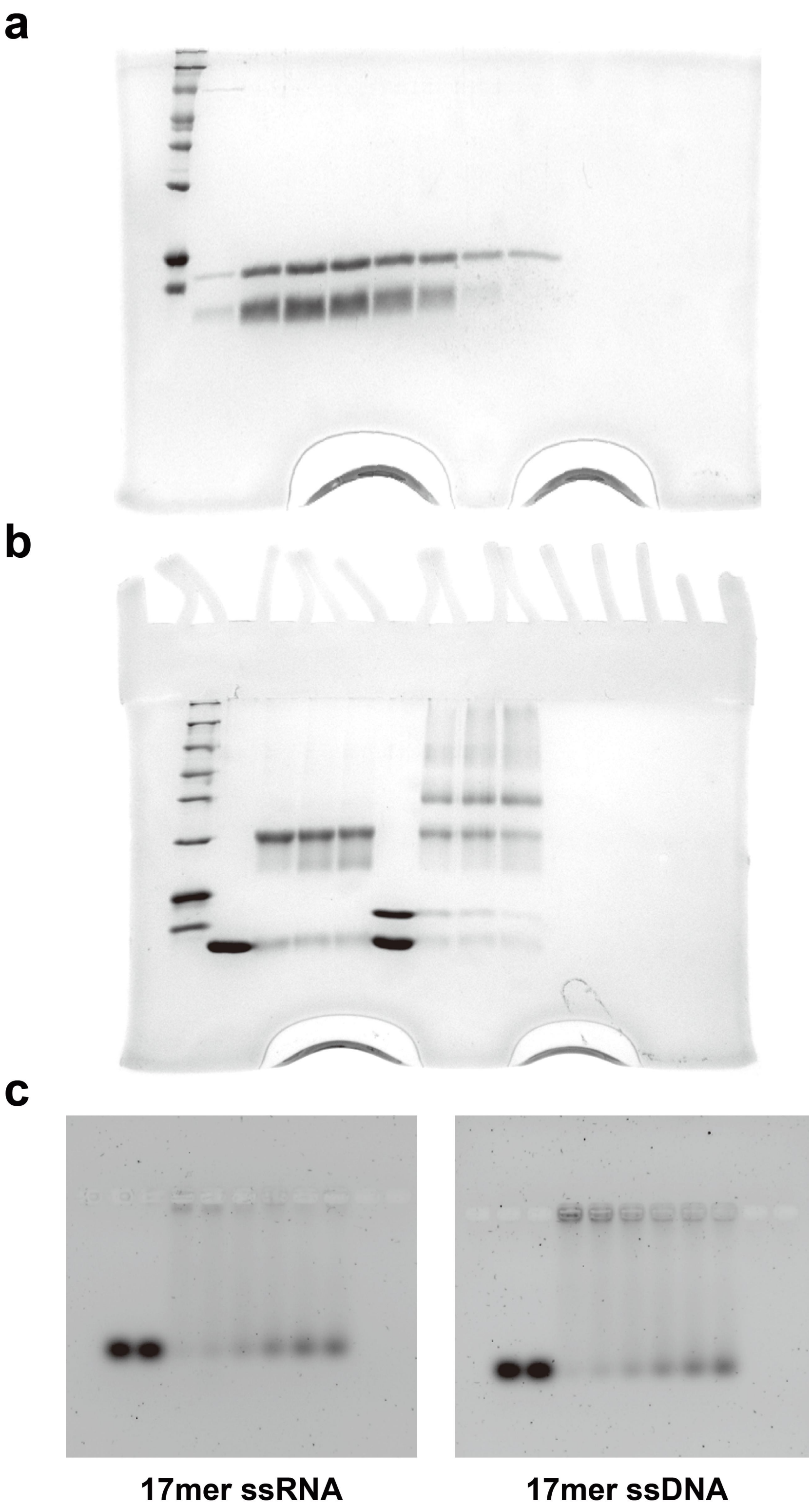

Supplementary Fig. 6. Uncropped Gels. a Unedited gel used in Figure 3a. **b** Unedited gel used in Figure 3d. **c** Unedited gels used in Figure 6a.

Supplementary Table 1. Primers used for SARS-CoV-2 *N*, *Nsp3* and *truncated Nsp3* in the fluorescence assay.

Gene	Forward primer (5′ to 3′)	Reverse primer (5′ to 3′)
N protein (1–419)	ACTCACTATAGGGAGACCCATGTCTGATAATGGAC	CGTATGGGTACCCGGGCATGGCCTGAGTTGAGTCAGCA
Nsp3.1 (1–654)	TCTATATAAGCAGAGCTCGTATGGCGCCGACCAAAGTGA	CGGCACCTTCAGGCTACGCATGTAACGAGCCGCTTCC
Nsp3.2 (649–1304)	GAGCTCCTTATGGCCTCTAGACGTAGCCTGAAGGTGCCG	CAGGGTTTTCAGACCCAGCACACGGCTCAGCTCGTT
Nsp3.3 (1299–1945)	CGAGCTCCTTATGGCCTCTAGACTCGAGGGTGGAAAGCTTCTGGGTCTGAAAACCCTG	GGAATTCGGCCACCCTTCAGAGCGAT
$Nsp3^{\Delta 111}$ (112–645)	TCTATATAAGCAGAGCTCGTATGGACGAGGAAGAGGGTGAC	CGGCACCTTCAGGCTACGCATGTAACGAGCCGCTTCC
$Nsp3^{\Delta 168}$ (169–645)	TCTATATAAGCAGAGCTCGTATGACCGTGGGCCAGCAGGAT	CGGCACCTTCAGGCTACGCATGTAACGAGCCGCTTCC
Ubl1 (1–111)	TCTATATAAGCAGAGCTCGTATGGCGCCGACCAAAGTGA	CTTGCTCACCAGAATTCGTTCATCCGGGTAGAAG

Supplementary Table 2. Primers used for SARS-CoV-2 *N*, *N truncations* and *Ubl1*.

Gene	Forward primer (5′ to 3′)	Reverse primer (5′ to 3′)
N protein (1–419)	CTAGCTAGCGAAAACCTGTATTTTCAGGGCATGTCTGATAATGGACCCCAAAATC	CGCGGATCCTTAGGCCTGAGTTGAGTCAGCACTGC
N-arm–NTD–LKR (1–247)	CTAGCTAGCATGTCTGATAATGGACCCCAAAATC	CGCGGATCCTTAAGTGACAGTTTGGCCTTGTTG
NTD–LKR (47–247)	CTAGCTAGCAATAATACTGCGTCTTGGTTCACCGC	CGCGGATCCTTAAGTGACAGTTTGGCCTTGTTGTTG
NTD (47–174)	CTAGCTAGCAATAATACTGCGTCTTGGTTCACC	CGCGGATCCTTATTCTGCGTAGAAGCCTTTTGG
CTD–C-tail (248–419)	CTAGCTAGCAAGAAATCTGCTGCTGAGGCTTC	CCGCTCGAGTTAGGCCTGAGTTGAGTCAGCAC
Ubl1 (1–111)	CGCCATATGGCGCCGACCAAAGTGACCTTCGGTG	CCGCTCGAGTTATTATTCATCCGGCGGGTAGAAGCTGCAGTAC

Supplementary Table 3. Primers used for SARS-CoV-2 *NTD* and *Ubl1* mutations.

Gene	Forward primer (5′ to 3′)	Reverse primer (5′ to 3′)
NTD R92A	TACCGAAGAGCTACCGCGCGAATTCGTG	CGCGGTAGCTCTTCGGTAGTAGCCAAT
Ubl1 E26A	GAGCGTGAACATCACCTTCGCGCTGGATGAACGTAT	TGTCGATACGTTCATCCAGCGCGAAGGTGATGTTCA
Ubl1 E95A	TTCGATGAGAGCGGCGCGTTCAAGCTGG	CGCGCCGCTCTCATCGAACAGGTAGTAG
Ubl1 Y103A	CAAGCTGGCGAGCCACATGGCCTGCAGCTTCTACCC	CCGGCGGGTAGAAGCTGCAGGCCATGTGGCTCGCCA
Ubl1 D110A	CGCCATATGGCGCCGACCAAAGTGACCTTCGGTG	CCGCTCGAGTTATTCCGCCGGCGGGTAGAAGCTGCAG

References

1. Gouet, P., Courcelle, E., Stuart, D. I., & Métoz, F. ESPript: analysis of multiple sequence alignments in PostScript. *Bioinformatics* **15**, 305–308 (1999).
2. Bessa, L. M. et al. The intrinsically disordered SARS-CoV-2 nucleoprotein in dynamic complex with its viral partner nsp3a. *Sci. Adv.* **8**, eabm4034 (2022).
3. Dinesh, D. C. et al. Structural basis of RNA recognition by the SARS-CoV-2 nucleocapsid phosphoprotein. *PLoS Pathog.* **16**, e1009100 (2020).
